# Diagnostic delay of myositis: an integrated systematic review

**DOI:** 10.1186/s13023-022-02570-9

**Published:** 2022-11-21

**Authors:** Tergel Namsrai, Anne Parkinson, Anita Chalmers, Christine Lowe, Matthew Cook, Christine Phillips, Jane Desborough

**Affiliations:** 1 National Centre for Epidemiology and Population Health, Australian National University, Canberra, ACT Australia; 2 The Myositis Association- Australia Inc, Berry, NSW Australia; 3 John Curtin School of Medical Research, Australian National University, Canberra, ACT Australia; 4 School of Medicine and Psychology, Australian National University, Canberra, ACT Australia

**Keywords:** Myositis, Idiopathic inflammatory myositis, Diagnostic delay, Systematic review, Meta-analysis

## Abstract

**Background:**

Idiopathic inflammatory myopathies (IIM) are a heterogenous group of rare muscular autoimmune diseases characterised by skeletal muscle inflammation with possible diagnostic delay. Our aim was to review the existing evidence to identify overall diagnostic delay for IIM, factors associated with diagnostic delay, and people’s experiences of diagnostic delay.

**Methods:**

Three databases and grey literature sources were searched. Diagnostic delay was defined as the period between the onset of symptoms and the year of first diagnosis of IIM. We pooled the mean delay using random effects inverse variance meta-analysis and performed subgroup analyses.

**Results:**

328 titles were identified from which 27 studies were included. Overall mean diagnostic delay was 27.91 months (95% CI 15.03–40.79, I^2^ = 99%). Subgroup analyses revealed a difference in diagnostic delay between non-inclusion body myositis (IBM) and IBM types. There was no difference in diagnostic delay between studies in which myositis specific autoantibodies (MSA) were tested or not tested. In countries with gatekeeper health systems, where primary care clinicians authorize access to specialty care, people experienced longer periods of diagnostic delay than people with IIM in countries with non-gatekeeper systems. While studies discussed factors that may influence diagnostic delay, significant associations were not identified. No qualitative studies examining people’s experiences of diagnostic delay were identified.

**Conclusion:**

Diagnostic delay of IIM has extensive impacts on the quality of life of people living with this disease. Understanding the experiences of people with IIM, from symptom onset to diagnosis, and factors that influence diagnostic delay is critical to inform clinical practice and training activities aimed at increasing awareness of this rare disease and expediting diagnosis.

*Trial registration*: PROSPERO Registration number: CRD42022307236 *URL of the PROSPERO registration*: https://www.crd.york.ac.uk/PROSPEROFILES/307236_PROTOCOL_20220127.pdf

**Supplementary Information:**

The online version contains supplementary material available at 10.1186/s13023-022-02570-9.

## Introduction

Idiopathic inflammatory myopathies (IIM) commonly described as “inflammatory myositis”, refers to a heterogenous group of rare muscular diseases characterised as skeletal muscle inflammation and other extra muscular features such as skin manifestations [1]. Subtypes of IIM including dermatomyositis (DM), polymyositis (PM), inclusion body myositis (IBM) and other specified types of idiopathic myositis (i.e. immune-mediated necrotising myopathy (IMNM/NM), juvenile myositis (JM), juvenile dermatomyositis (JDM), amyopathic dermatomyositis (AMD) and anti-synthetase syndrome (ASS)), and unspecified idiopathic inflammatory myositis) [2]. IIM has broad clinical characteristic features involving both muscular and extra-muscular systems, with acute or progressive onset. In addition to general muscle features, it can present with dysphagia (39%), interstitial lung disease (ILD) (30%), malignancy (13%), and cardiac disease (9%) [3].

In the last decade there has been promising progress in identifying myositis specific autoantibodies (MSA), with 95% specificity but only 20% sensitivity in diagnosis of IIM [4]. More recently, Dalakas introduced different criteria which takes into account AMD [5]. However, these two sets of criteria both still exclude IBM as an individual type of IIM. In 2017, the European League Against Rheumatism (EULAR) and the American College of Rheumatology (ACR) developed diagnostic and classification criteria based on data from 976 IIM cases and 624 comparators [6]. The EULAR/ARC criteria enable clinicians to differentiate between all possible IIM subgroups that are not mentioned in previously used criteria, including JM, JDM and IMNM. Similarly, other criteria developed by the European Neuromuscular Centre (ENMC) incorporate all subtypes of IIM and are used alongside the other criteria [7].

Due to the low prevalence of IIM, range of clinical features, lack of comprehensive and internationally accepted diagnostic criteria, diagnosis of IIM can be challenging and many patients experience significant diagnostic delays. Some studies have reported diagnostic delay of 4–5.6 years in cases of IBM [8, 9]. However, studies examining the overall diagnostic delay, factors associated with diagnostic delay, and people’s experiences of diagnostic delay in IIM are scarce. Our aim was to systematically review the evidence about diagnostic delay in IIM to provide insight into time to diagnosis, factors associated with diagnostic delay, and people with myositis’ experiences of diagnostic delay. This may be used to inform the development of interventions, tools, and health policies directed at enhancing diagnostic efficiency of IIM.

## Methods

We performed a systematic review and reported in accordance with the Preferred Reporting Items for Systematic Reviews and Meta-Analyses (PRISMA) and and the Cochrane Handbook for Systematic Reviews [10, 11]. This review is registered with PROSPERO, an international prospective register of systematic reviews (registration number: CRD42022289830). The protocol for this review is provided in Additional file 1.

### Search, study selection, and data extraction

Searches of three electronic databases: PubMed/Medline, Scopus, and ProQuest were conducted on the 9th of December 2021 using the search string: Myositis AND (“delay in diagnosis” OR “diagnostic delay” OR “misdiagnosis” OR “time to diagnosis” OR “incorrect diagnosis” OR “missed diagnosis” OR “delayed diagnosis”). The final search strategy that was developed and used on the  Pubmed/Medline database is shown in Additional file 1: Table 1. Grey literature searches were conducted from 9 to 15th December 2021: Open Access Theses and Dissertations (https://oatd.org/), ProQuest thesis and dissertations, the National Library of Australia, and the Myositis Association Australia website (https://myositis.org.au/). Manual reference searches were conducted on all review articles found by literature search.

No restriction on the publication date was applied. All study types (qualitative and quantitative) except review articles, examining diagnostic delay, incorrect diagnosis, missed diagnosis or slow diagnosis of all types of myositis in all age groups were included. Studies in languages other than English, German and Indonesian were excluded. Search results were imported into Covidence, an internet-based software that facilitated collaboration between reviewers [12].

Two authors (AP, TN) independently screened titles and abstracts, and then the full-text articles of the remaining studies were screened against pre-developed PICOS eligibility criteria as outlined in Table 1. Articles with population, exposure, or outcome other than that outlined in PICOS were excluded. At each stage, discrepancies were resolved through discussion moderated by a third reviewer (JD). The data extraction tool (Additional file 1: Table 2) was developed and peer-reviewed by the research team, and independently piloted (AP, TN) on five studies. One author (TN) then extracted the data using this tool.

**Table 1:** PICOS eligibility criteria

PICOS	Inclusion criteria	Exclusion criteria
Population	Studies examining people of all ages with myositis including dermatomyositis, polymyositis, necrotising myositis, juvenile dermatomyositis, inclusion body myositis, mixed connective tissue diseases, overlap myositis, interstitial myositis, orbital myositis and antisynthetase syndrome	–
Intervention/Exposure	Studies examining delayed, incorrect diagnosis, missed diagnosis or slow diagnosis of myositis	–
Comparison	Not applicable	–
OUTCOME	The primary outcome is diagnostic delay. Probable secondary outcomes are causes and consequences of diagnostic delay and patients’ experiences of diagnosis of myositis	–
Study design	All study designs	Review articles
Language	English, German, Indonesian	
Setting	No restriction	–
Timing	No restriction	–

### Quality appraisal

**Fig. 1:**
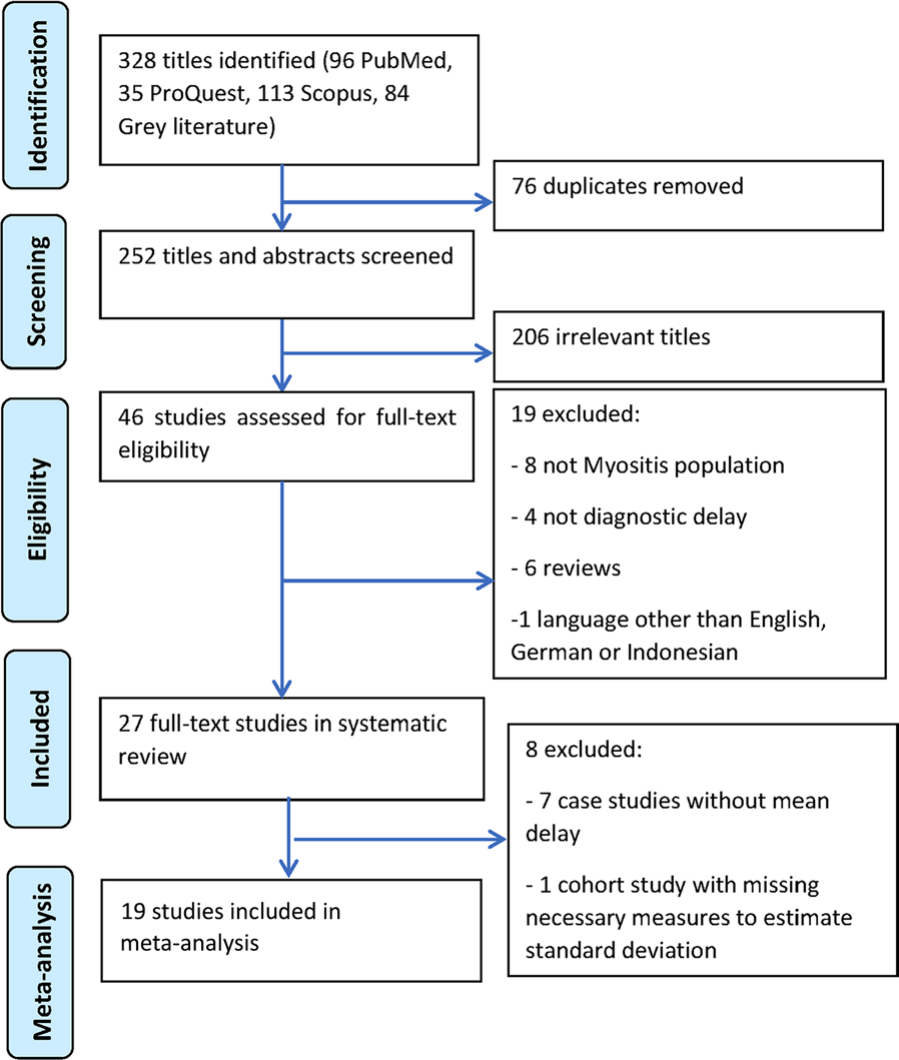
Study selection flow chart

Studies were assessed for risk of bias using adapted versions of the Newcastle–Ottawa Scale [13] summarised in Additional file 1: Table 3 and Fig. 1. The highest possible score in the adapted version of the Newcastle–Ottawa scale is seven which represents the lowest risk of bias. Overall, two studies received a score of seven [14, 15], five studies scored six [9, 16–19], eight studies scored five [19–26], three studies scored four [27–29], while nine studies scored equal or less than three which represents a higher risk of bias [30–38].

### Data analysis

We defined diagnostic delay in accordance with the studies [e.g., time from reported onset of symptoms to definitive diagnosis]. The primary outcome of mean diagnostic delay, presented in years and months in all studies, was converted to months for all studies. For studies examining ASS, only data describing mean delay for the complete form of ASS were extracted, as complete ASS is characterised as arthritis, ILD, and myositis. Mean delay in months was pooled using inverse variance weighted random effects models (DerSimonian–Laird method). Where the standard deviation (SD) of diagnostic delay was missing, we imputed it using the method recommended by Cochrane, which calculates the SD using upper limit, lower limit and the confidence interval [39]. When confidence intervals were missing, the SD was calculated using a method improved by Wan and others which incorporated the sample size or population [40]. Sensitivity analyses between non-SD estimated studies and SD estimated studies were conducted. Additionally, subgroup analyses between (1) different IIM types, (2) non-IBM versus IBM groups, (3) MSA tested versus MSA not tested, (4) gatekeeper health system versus non-gatekeeper health system, (5) Peter Bohan’s versus ENMC criteria (including versions from 1997, 2003 and 2011), and (6) multidisciplinary centres versus specialist centres were conducted. Studies that reported the testing of one or more antibodies from MSA were considered MSA tested. Studies conducted in multiple countries were not included in the fourth subgroup analysis due to inability to determine the dominant health system used. Studies using records of only biopsy, both clinical features and biopsy were excluded. Additionally, studies that investigated ASS were excluded as criteria for ASS is different from other IIM subtypes. After excluded studies, five studies that used either Peter and Bohan’s criteria or ENMC criteria were left. Studies located in treatment/diagnosis centres were identified where authors mentioned “at our centre” or the name of the centre and were checked against affiliations/contact details. Multidisciplinary centres were defined as centres with multiple disciplines including university hospitals and tertiary centres while specialist centres were defined as special-focus centres that are dedicated to IIM, including members of the Australasian Neuromuscular Network. One study was conducted at an ‘other centre' (i.e. departments other than multidisciplinary or specialist centres) and was excluded from the subgroup analysis. Heterogeneity of meta-analysis estimates was presented using the I^2^ statistic. Funnel plots were used to assess the risk of publication bias. We extracted further data from the included studies to identify the significance of the factors potentially associated with diagnostic delay (initial specialist, initial symptoms, symptoms that changed the diagnosis, muscle biopsy status, creatinine kinase levels, treatment/diagnosis centres), outcomes of diagnostic delay, and people’s lived experiences of diagnostic delay. When possible, meta-aggregation was conducted to focus on meanings from qualitative data and aggregate them into categories with similar meanings that could be synthesised and analysed. Analyses were performed using R version 4.6.2 (R Foundation for Statistical Computing, Vienna, Austria) and the ‘meta’ package.

## Results

Out of 328 studies identified, 76 duplicates were removed and 206 were excluded at title and abstract screening. A further 19 studies were excluded at full-text screening. The remaining 27 studies published between 1992 and 2020 were included in the review as described in Fig. 1.

### Description of included studies

The descriptive summary of the selected studies is presented in Table 2. A full data extraction table is shown in Additional file 1: Table 4. The 27 studies included in the review consisted of seventeen non-comparative studies (nine descriptive cross-sectional studies [9, 14, 16, 17, 22, 23, 29, 36, 38], seven case reports [27, 28, 31–35] and one case series [30]), six analytical cross-sectional studies [15, 18–20, 24, 37], one time series with comparison group [21] and three retrospective cohort studies [25, 26, 41]. Studies were from multiple countries, including nine from Europe [9, 15, 16, 25, 27, 31, 35, 37, 38], eight from the United States [20, 21, 24, 29, 30, 33, 34, 41], three from Australia [19, 22, 23], one from New Zealand [14], two from Asia [28, 32], and one from South America [26]. Two studies were multi-national [18, 36]. Studies reported diagnostic delay in all IIM subtypes; DM [28, 29, 33] (n = 3 studies), IBM [9, 14, 16, 17, 21–24, 27, 30, 34–36] (n = 13 studies), ASS [18, 26, 31, 32] (n = 4 studies), JDM [15, 20] (n = 2 studies), NM [41] (n = 1 study) and mixed or all types of IIM [19, 21, 25, 38] (n = 4 studies). In total, based on the IIM subtypes, the systematic review included a sample size of 1827 with the highest sample size for IBM (n = 1262 people) [9, 14, 16, 21–24, 27, 30, 34–36] and the lowest for NM [41] (n = 67 people).

**Table 2:** Descriptive summary table of selected studies (n = 27)

Author	Country	MSA† Tested	Study sample (n)	Mean age (year)	Type of IIM studied	Diagnostic approach/diagnostic criteria used	Mean delay (months)	Mean delay SD^⁂^ (months)
*Retrospective cohort studies*								
Baccaro et al. [26]	Brazil	Yes	55	Not reported	ASS	Criteria proposed by Connors et al. and Cavagna et al. [51, 52]	29	8.99
Cobo-Ibanez et al. [25]	Spain	Yes	478	47.7	All types of IIM	Peter and Bohan’s criteria	3.48	7.85
Triplett et al. [41]	United States	Yes	67	Not reported	NM	Biopsy and electromyography	8.5	28.04
*Time series with comparison group*								
Sayers et al. [21]	United States	No	32	61	IBM	Peter and Bohan’s criteria	34	24.7
*Analytical cross-sectional studies or time series*								
Cavagna et al. [18]	Multi-national	Yes	44	53.5	ASS	Clinical characteristics and positive anti Jo-1	5	6.51
Kazamel et al. [24]	United States	No	51	Not reported	IBM	Grigg's pathological criteria [53]	74.4	75.67
Mathiesen et al. [15]	Denmark	No	57	Not reported	JDM	Peter and Bohan’s criteria	8	1.6
Pijnenburg et al. [37]	France	No	40	48.2	All types of IIM	ENMC criteria	16.4	4.5
Wargula et al. [20]	United States	No	59	7.9	JDM	Peter and Bohan’s criteria	5.3	5.2
Williams et al. [19]	Australia	No	13	68	All types of IIM	Serum creatinine kinase level, electromyography, and biopsy	55	53.49
*Non-comparative studies (descriptive cross-sectional studies, survey and prevalence or incidence studies)*								
Badrising et al. [16]	The Netherlands	No	76	Not reported	IBM	ENMC criteria of 1997	96	71.24
Da Silva et al. [29]	United States	No	232	Not reported	DM	Not reported	15.5	46.61
Dobloug et al. [9]	Norway	Yes	100	Not reported	IBM	ENMC criteria of 1997 or 2011	67.2	60
Felice et al. [17]	United States	No	35	70	IBM	Definite or possible IBM as proposed by Griggs et al. [53]	68.4	68.47
Lynn et al. [14]	New Zealand	No	6	Not reported	IBM	Peter and Bohan’s criteria/Mastaglia and Phillips [54]	43.2	10.3
Needham et al. [23]	Australia	No	57	Not reported	IBM	Needham and Mastaglia’s criteria [55]	62.4	39.24
Paltiel et al. [36]	Multi-national	No	280	70.4	IBM	Not reported	56.4	Not reported
Phillips et al. [22]	Australia	No	17	Not reported	IBM	Grigg's pathological criteria [53]	52.8	26.72
Rotar et al. [38]	Slovenia	No	79	Not reported	All types of IIM	Records of biopsy	6.66	6.77
*Non-comparative studies (Case reports)*								
De Langhe et al. [31]	Belgium	Yes	1	44	ASS	Clinical characteristics and presence of antisynthetase antibodies	48	Not calculatable
Devi et al. [32]	India	Yes	1	35	ASS	Not reported	0	Not calculatable
Dickison et al. [33]	United States	Yes	1	31	DM	Clinical characteristics and biopsy	120	Not calculatable
Herath et al. [28]	Sri Lanka	Yes	1	53	DM	Peter and Bohan’s criteria	5	Not calculatable
Hom et al. [34]	United States	No	1	58	IBM	Biopsy	60	Not calculatable
Kucuksen et al. [27]	Turkey	No	1	63	IBM	Biopsy	60	Not calculatable
Munshi et al. [35]	UK	No	1	81	IBM	Biopsy	36	Not calculatable
*Non comparative studies (Case series)*								
Chilingaryan et al. [30]	United States	No	20	67.8	IBM	Not reported	70	54.8

^**†**^MSA- myositis specific antibody test

^**⁂**^SD- Standard deviation

### Pooled diagnostic delay in IIM

**Fig. 2:**
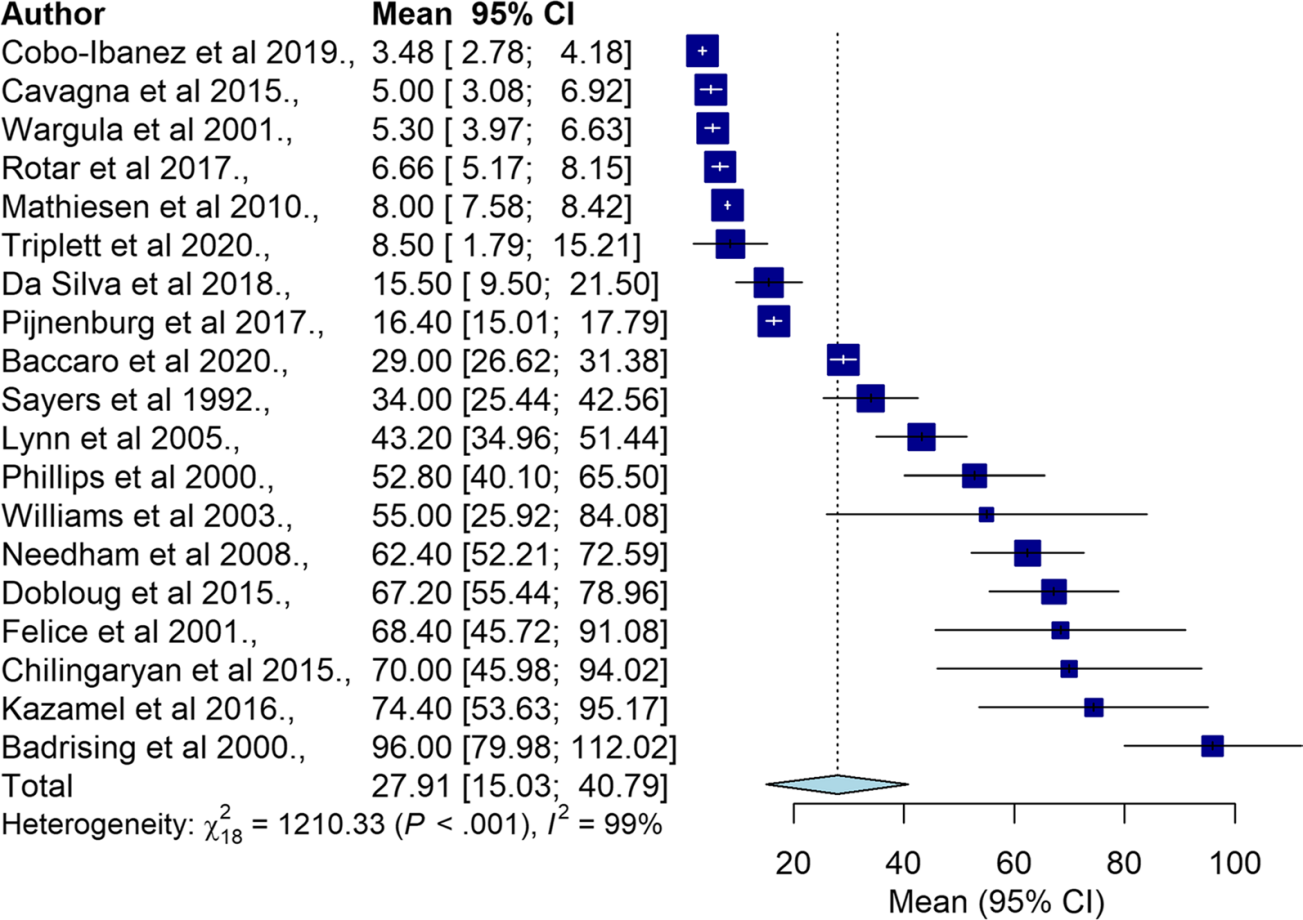
Forrest plot for mean diagnostic delay in all types of IIM

Nineteen studies were included in the meta-analysis with a total sample size of 1518 people. Individual study sample size for the 19 studies range from six [14] to 478 [25]. The mean diagnostic delay in IIM ranged from 3.48 [25] to 96.0 months [16]. The pooled overall mean diagnostic delay was 27.91 months (95% CI 15.03–40.79, I^2^ = 99%) as outlined in Fig. 2. The funnel plot for overall mean diagnostic delay is shown in Additional file 1: Figure S2. Excluding studies with estimated SD showed similar results as summarised in Additional file 1: Figure S3.

### Subgroup analyses

**Fig. 3:**
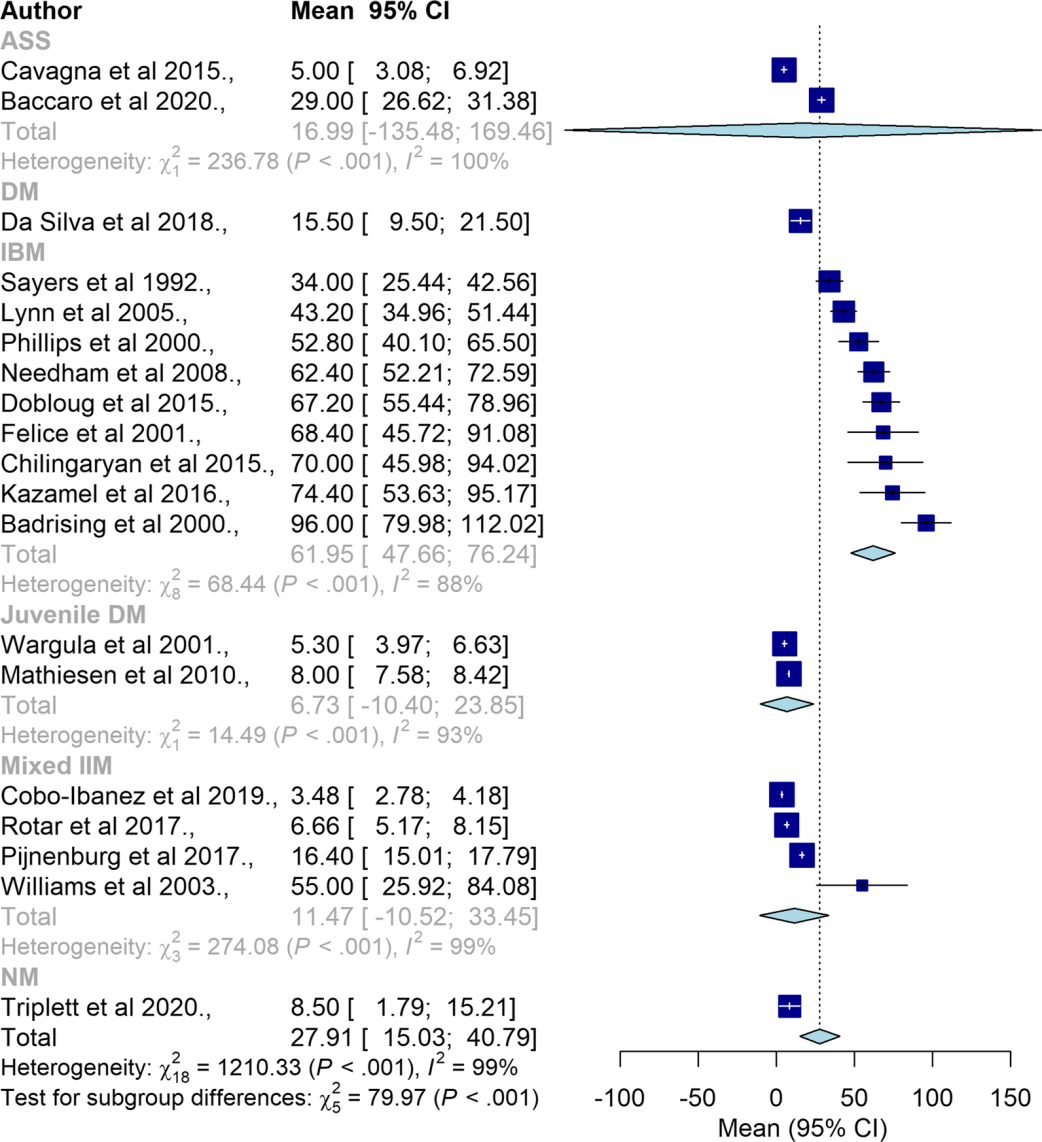
Forrest plot for all types of IIM

Subgroup analyses between different types of IIM is summarised in Fig. 3 (n = 19). Subgroup analyses revealed significant differences in mean diagnostic delay between different IIM subtypes. Compared to other IIM subtypes JDM had the shortest mean delay (6.73 months, 95% CI = − 10.40–23.85) whereas IBM had the longest mean delay (61.95 months, 95% CI = 47.66–76.24).

**Fig. 4:**
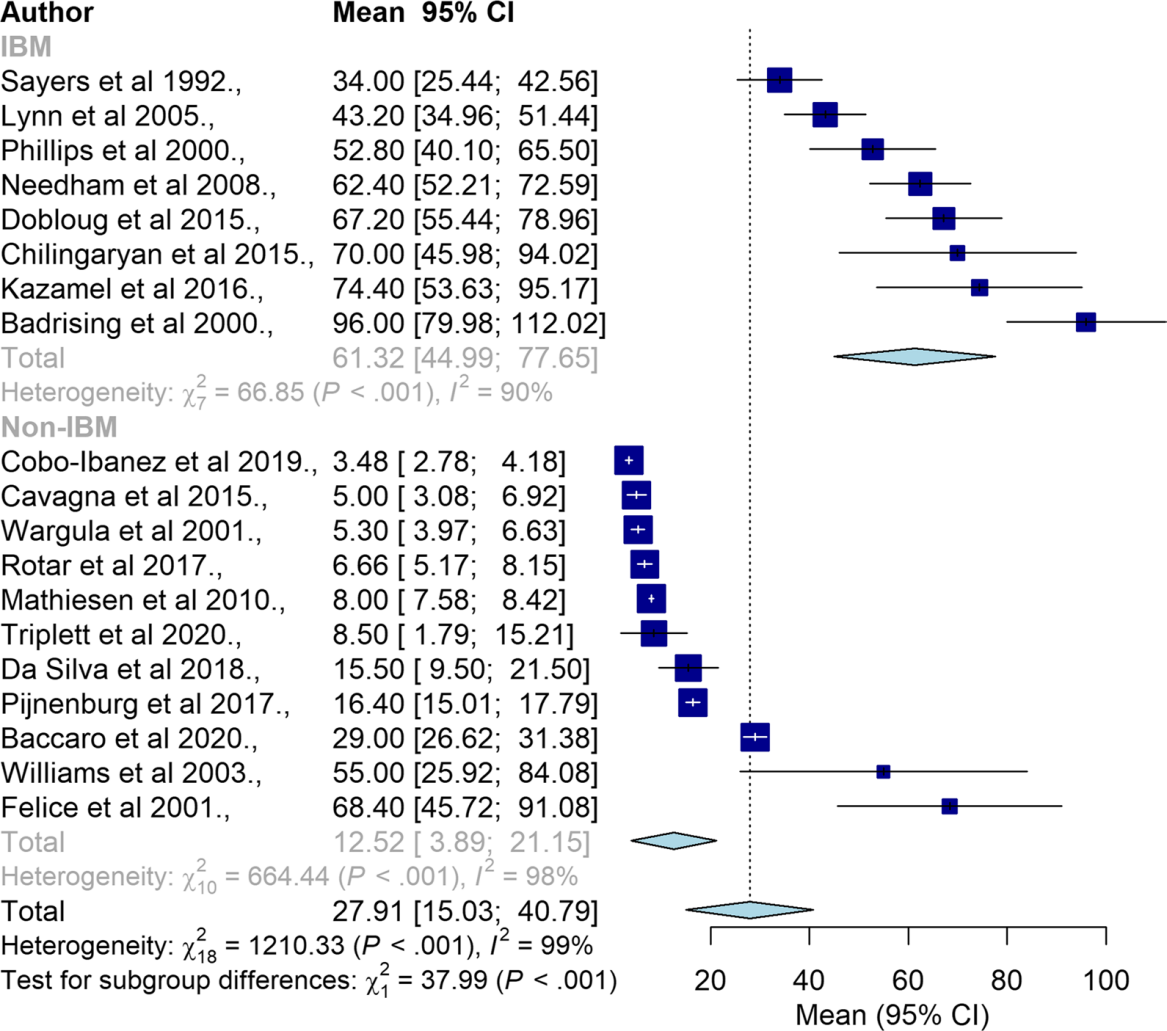
Forrest plot for mean diagnostic delay in IBM and non-IBM types of IIM

Subgroup analyses between IBM and non-IBM types are summarised in Fig. 4 (n = 19). There was a significant difference in mean diagnostic delay between IBM and non-IBM types. Compared to non-IBM (12.52 months, 95% CI = 3.89–21.15), IBM type had significantly longer mean diagnostic delay (61.32 months, 95% CI = 44.99–77.65).

Subgroup analysis between studies for which MSA was tested versus studies for which MSA was not tested is presented in Additional file 1: Figure S4 (n = 19). The mean diagnostic delay did not differ between ‘MSA-tested’ and ‘MSA-not-tested’ studies.

**Fig. 5:**
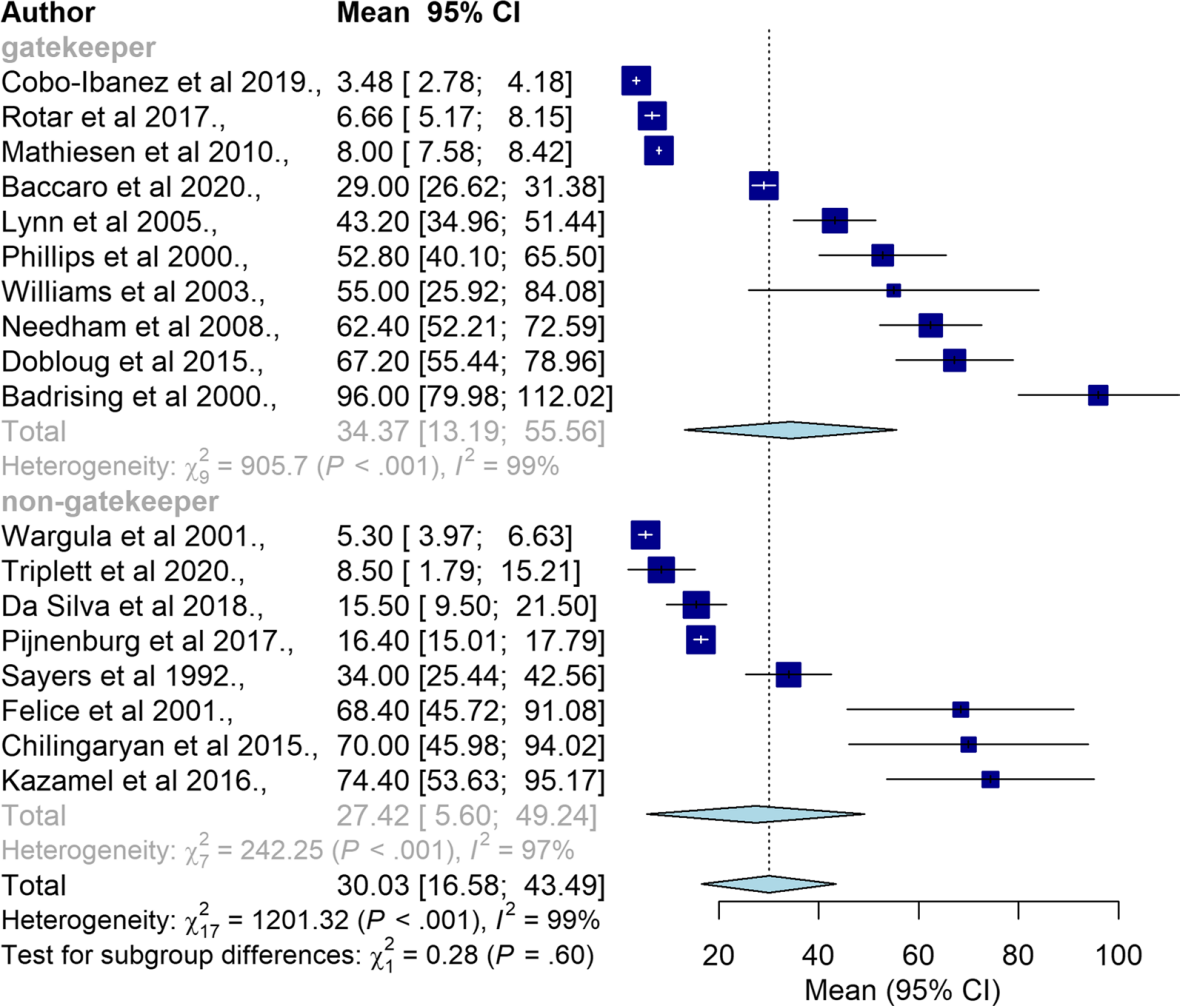
Subgroup analysis between gatekeeper and non-gatekeeper health systems

Subgroup analysis between studies conducted in gatekeeper health systems versus non-gatekeeper health systems is summarised in Fig. 5 (n = 18). Countries with a gatekeeper health system had significantly longer diagnostic delay (34.37 months, 95% CI = 13.19–55.56) compared to countries with a non-gatekeeper health system (27.42 months, 95% CI = 5.60–49.24).

Subgroup analysis between studies that used Peter and Bohan’s criteria and ENMC criteria is presented in Additional file 1: Figure S5 (n = 5). There was no significant difference in mean diagnostic delay in studies where Peter and Bohan’s criteria and those where the ENMC criteria was used.

Subgroup analysis comparing studies at multidisciplinary centres and specialist centres is presented in Additional file 1: Figure S6 (n = 16). The mean diagnostic delay did not differ between different centres.

### Case studies

There were eight case studies, including seven case reports [27, 28, 31–35] and one IBM case series [30]. The seven case reports consisted of three IBM [27, 34, 35], two ASS [31, 32], and two DM case studies [28, 33]. These included an overall sample size of 27 people (17 males, 10 females, mean age = 56.41 years, (95% CI = − 73.68 to 186.60)). Mean diagnostic delay in case studies was 49.87 months (95% CI = 17.92–81.83) ranging from 0 months for ASS [32] and 120 months for DM [33].

### Initial specialist, initial symptoms, most common symptoms, and symptoms that changed the diagnosis

Four of the included studies reported the initial specialist seen by patients [17, 30–32] as described in Additional file 1: Table S4. Due to the lack of available data, further analysis on the difference in diagnostic delay related to the initial specialist seen could not be conducted.

Eighteen of the included studies reported a broad range of initial symptoms as shown in Additional file 1: Table S5. Due to heterogenous data, investigation of associations between the initial symptoms and the diagnostic delay was not conducted. However, further analysis of initial symptoms with meta-aggregation by each subtype of IIM is presented in Additional file 1: Table S5. Based on the case reports, 50% (3/6) of the reported symptoms for ASS were lung symptoms, and for IBM 83.3% of initial symptoms were muscle associated symptoms (5/6 symptoms). In JDM, based on a single time series [18] and one cross-sectional study [20], the most common initial symptoms were muscle symptoms (proximal muscle weakness (93%), muscle pain (31–75%)), skin symptoms (Gottron’s papules (74%), heliotrope rash (67%), periungual/nailfold capillary changes (35–75.6%), malar erythema (48%)), and general symptoms including (fatigue (44–82%), weight loss (33–44%), and arthralgia (40–61%)).

Nine studies consisting of two retrospective cohort studies [21, 25], five cross sectional studies [15, 18, 20, 24, 37], and two non-comparative descriptive studies [9, 29] reported the most common symptoms of their study samples. The most frequently-reported symptom was muscle weakness, reported in 8/9 studies, particularly proximal muscle weakness [9, 15, 20, 21, 37].

All case studies reported the symptom that changed the diagnostic approach [27, 28, 30–35], which in 6/8 described progression of symptoms [27, 28, 30, 31, 34, 35] (refer to Additional file 1: Table S4). In these cases, symptoms progressed to dyspnoea, fever, night sweats, weight loss, myalgia, muscle weakness, arthralgia, swollen eyelids and cracked fingers [31], recurrent falls [35], dependence on assistance with daily living activities [27], extreme loss of weight [34], extreme poikiloderma on 90% of body and and inability to walk, climb stairs and get up from seated position [30]. In addition to disease progression, one study also described new symptoms [28]. The appearance of new symptoms alone changed the diagnostic approach in one study [33]. New symptoms included heliotrope rash, Gottron’s papules [28] and erythematous patches [33]. In one case, no response to treatment changed the diagnostic approach [32]. Half of the case studies reported treatment after misdiagnosis [28, 32–34]; these included antibiotics in three cases [28, 32, 33] and occupational therapy in one case [34].

### Muscle biopsy and serum creatine kinase (CK) levels

Twenty-one studies confirmed conducting muscle or tissue biopsy as part of the diagnostic approach or as part of an inclusion criteria as shown in Additional file 1: Table S4 [9, 14–17, 19–24, 27, 28, 30, 33, 35–38, 41]. However, there was inadequate data to investigate the presence of correlations or associations between muscle biopsy status and diagnostic delay in IIM.

Twelve studies reported mean CK levels or individual CK levels (case reports) (Additional file 1: Table S4). Six case studies [27, 28, 31, 32, 34, 35] reported individual CK levels of which three (50%) found elevated levels [27, 31, 35]. One case series reported CK levels for two cases (2/20); raised levels were detected for both [30]. Five studies with multiple samples reported increased mean CK levels [9, 14, 15, 17, 21] ranging from 444 [17] to 3589 U/L [14]. However, due to lack of data availability mean pooled CK levels and its association with diagnostic delay could not be evaluated.

### Factors related to diagnostic delay

While case studies are not designed to examine the association between diagnostic delay and symptoms of IIM, 10/27 studies (four retrospective cohort studies [15, 17, 23, 26], one cross sectional study [19], five case reports [28, 31, 33–35] and one case series [30]) mentioned 15 possible factors related to diagnostic delay in IIM as outlined in Additional file 1: Table S6. Two factors were related to health care service, 4/15 were clinician related factors [15, 23, 35] and 8/15 factors were related to the complex clinical characteristics of IIM [17, 19, 26, 28, 30, 31, 33, 34] while one was related to the patient’s lack of awareness of the severity of symptoms [23] (muscle weakness was thought to be due to normal ageing). None of the included studies examined factors associated with diagnostic delay. One study found that patient’s delays (time from symptom onset to first visit to a neurologist or rheumatologist) were longer than doctor’s delays (time from first visit to diagnosis) [16].

Analysis of diagnostic delay in relation to IIM subtypes is presented in Additional file 1: Table S7. The presence of complex clinical characteristics that were found to contribute to diagnostic delay were reported in relation to ASS (n = 2) [26, 31], DM (n = 2) [28, 33], IBM (n = 6) [19], and mixed IIM (n = 1) [21]. Clinician related factors that contributed to diagnostic delay were identified in relation to IBM (n = 3) [25, 32, 37], JDM (n = 1) [9], and one study reported a health care service related factor for IIM (n = 2) [21].

### Outcomes and experiences related to diagnostic delay

Sixteen studies mentioned outcomes or experiences of diagnostic delay (five retrospective cohort studies [15, 17, 23, 25, 29], two cross sectional studies [16, 36], one case control study, seven case reports [27, 28, 31–35] and one case series [30]). Nine of the 16 studies reported several misdiagnoses including motor neurone disease [16, 17, 23, 30], myopathy [16, 17], facioscapulohumeral muscular dystrophy [17], oculopharyngeal muscular dystrophy [17], peripheral nerve disease [17], polyneuropathy[16], entrapment neuropathy [30], Parkinson’s disease, lupus [29], undifferentiated connective tissue disease [29, 31], arthritis [23], old age [23], pneumonia [32] and bacterial vaginitis [33]. Six of the 16 studies reported worsening outcomes of disease as symptoms progressed, including extreme weight loss [34], more organ damage [15], increased need of assistance in daily living activities [27], recurrent falls [35], increased mortality [25] and increased camptocormia or dropped head syndrome [37]. Three case reports also referred to incorrect treatments [28, 32, 33] including antibiotics in three cases [28, 32, 33] and occupational therapy in one case [34]. In one case report, the patient was discharged early [28]. However, the effect of incorrect treatment or early discharge was not mentioned or studied.

Three of the 16 studies examined associations or correlations between disease outcomes and diagnostic delay. One retrospective cohort study found that shorter disease duration was correlated with less organ damage [15]. Another retrospective study found that delay in diagnosis was significantly associated with mortality in IIM with interstitial lung disease (HR 1.29, 95% CI 1.06–1.56) [25]. One case control study found delayed or longer IIM diagnosis in camptocormia and dropped head syndrome compared to the control group (without dropped head syndrome) [37].

None of the included studies used qualitative methods or examined people with myositis’ experiences of diagnostic delay.

## Discussion

The overall pooled mean diagnostic delay for IIM was (27.91 months or 2.25 years, 95% CI 15.03–40.79 months, I^2^ = 99%) and is similar to other rheumatologic diseases with long diagnostic delay [42]. Mean diagnostic delay varied greatly between IBM and non-IBM groups (61.32 months or 5 years [16, 29], 95% CI = 44.99–77.65, versus 12.52 months or 1 year, 95% CI = 3.89–21.15). The unique clinical characterisations of IBM could be one reason for the significantly longer diagnostic delay in IBM as it is the only IIM type that starts with slowly progressing asymmetric distal muscle weakness [1].

We found longer diagnostic delay in gatekeeper health systems when compared with non-gatekeeper health systems, indicating that the difference in accessibility to specialists influences time to diagnosis. A systematic review of the impacts of gatekeeping by general practitioners found that, compared to non-gatekeeper systems, gatekeeper systems result in better quality care and lower health care expenditure, but lower levels of patient satisfaction [43]. People with rare diseases such as IIM frequently present prior to diagnosis with symptoms that are seen frequently in general practice and are usually not attributable to a rare disease [44]. In these settings, clinicians need to consider more generally the presentation of a patient over time; common presentations that are unusual in intensity or periodicity may herald a rare disease.

There were no differences in diagnostic delay between studies where MSA was tested and those where MSA was not tested. This could be due to the small number of studies included in the MSA-tested group. It is also pertinent to consider the fact that studies with at least one of the tests were included in the MSA-tested group as none of the included studies conducted all MSA tests on their study population. Thus, incomplete testing of MSA could potentially result in the same amount of diagnostic delay. As MSA testing is a relatively new concept, it may take some time before it is embedded into practice and the impact of this on diagnostic delay is reflected in the literature.

Based on the current evidence, Peter and Bohan’s criteria has 94–98% sensitivity and 29–55% specificity while ENMC classification has 52–71% sensitivity and 82–97% specificity [45]. However, our subgroup analysis between Peter and Bohan’s criteria and ENMC criteria did not reveal any subgroup differences in diagnostic delay which could be due to the small study sample (n = 5 studies). This could also be due to the presence of other factors that could influence diagnostic delay including complex clinical characteristics, health care related factors, and clinician related factors. Due to a lack of studies using EULAR/ARC criteria to examine diagnostic delay, further analysis between Peter and Bohan’s criteria and EULAR/ARC criteria was not conducted. However, a recent validation study reported better subgroup classification with EULAR/ARC criteria compared to Peter and Bohan’s criteria [46]. Therefore, to clarify the impact of diagnostic criteria on diagnostic delay in IIM, further studies comparing the different types of diagnostic criteria and diagnostic delay are needed.

In the diagnosis of IIM, interdisciplinary centres and multidisciplinary centres play an important role as clinical features of IIM can be complex and require treatment from a multidisciplinary team. We explored the difference in diagnostic delay between multidisciplinary centres and specialist centres and did not find any significant difference. This may represent our own classification of these centres, which in practice may have been similar in nature. We are unable to comment on differences between multidisciplinary centres and single discipline specialist practice.

We found that most studies reported elevated CK levels despite CK being not specific to the diagnosis of IIM. Other factors may affect the CK level, including physical activity and other morbidities. Nevertheless, we suggest highly elevated CK levels may indicate an underlying inflammatory disorder, and may function as a red flag for clinicians to further explore the possibility of IIM if this is supported by physical symptoms and signs.

Several studies attempted to gain insight into diagnostic delay. One study reported overall diagnostic delay as either doctor’s delay (time from first visit to diagnosis) or patient’s delay (time from symptom onset to first visit to a neurologist or rheumatologist), and found that patient’s delays were longer. The study's authors proposed several factors to be related to diagnostic delay in IIM including those related to health care services or clinicians, and the complex clinical characteristics of IIM. Based on the factors analysed in each subtype of IIM we developed the following general insights to inform future improvements in relation to the diagnosis of IIM. Firstly, a focus on the evolving nature of a condition as experienced and reported by patients, with iterative recognition of emerging conditions, may help clinicians arrive at a diagnosis earlier. Symptoms of ASS can emerge at different time points. For example, pulmonary symptoms (dyspnoea) can present before symptoms of myositis [26, 31]. The classic skin signs of DM may be presaged by manifestations such as persistent vulvovaginitis and unexplained erythoderma and poikiloderma [28, 33]. Secondly, a holistic approach (combining patient history and diagnostic tests) should be used to support the appropriate use and interpretation of diagnostic tools and biopsy findings. IBM can present with a broad range of symptoms, amenable to a range of different diagnoses. The complex clinical features of IIM can include dysphagia without muscle weakness. Thirdly, clinicians should maintain an open mind to tests that “rule out” a potential diagnosis in the presence of ongoing symptoms. Among the many potential barriers to early diagnosis is that IBM can have atypical findings on muscle biopsy with normal electrophysiological findings; an over-reliance by clinicians on the need for atypia in both diagnostic modalities may result in missing some cases.

We did not identify any qualitative studies examining how people with myositis experienced diagnostic delay. Understanding people’s experiences from symptom onset until diagnosis may assist in elucidating factors influencing diagnosis and diagnostic delay in IIM. This may inform strategies aimed at raising awareness and the development of resources to support clinical reasoning and identify points in patients’ journeys when existing diagnoses should be re-evaluated, and a rare disease diagnosis considered.

Some outcomes of diagnostic delay were described, including disease progression, organ damage, deterioration in capacity to manage the activities of daily living and increased mortality. These outcomes highlight the critical need to improve awareness, understanding and diagnosis of IIM. All forms of myositis significantly impact the quality of life of those who are diagnosed, as they present with a broad range of debilitating symptoms requiring ongoing medical treatment. People with myositis report pain, fatigue, and day-to-day fluctuation of symptoms as being the most impactful symptoms [47–49]. Research examining people’s experiences with multiple sclerosis has found that delays in diagnosis may create a sense of uncertainty and, in many cases, a worsening of symptoms, leaving people in a state of ‘not knowing’ [50]. As their ability to participate in activities that give them pleasure becomes more limited and their ability to carry out daily activities is reduced, the emotional consequences of the disease may compound. One study of the experiences of people with myositis found that they greatly valued being able to discuss issues and concerns with their clinician(s) about their future and the potential impact of myositis on their quality of life, enabling them to plan and prepare [49]. Unfortunately, this cannot be addressed until a diagnosis is received and treatment regime determined.

To the best of our knowledge this is the first systematic review to examine the length of diagnostic delay in myositis. The key strength of this study is the inclusion of a large number of studies and relatively large sample size (n = 1827 people) representing all types of myositis. The main limitation is the need to estimate the standard deviation in 19 studies using the mean and range or interquartile range. However, the method used to estimate missing standard deviations is an improved method that incorporates the study population and provides nearly unbiased standard deviation of the true population [40].

## Conclusion

Diagnostic delay of IIM has extensive impacts on the quality of life of people living with this disease. There is lack of both qualitative and quantitative research examining people’s experiences of, and factors associated with, diagnostic delay in IIM. These studies are crucial to inform the development of tools and strategies aimed at increasing awareness of IIM and reducing diagnostic delay.

## Supplementary Information

**Additional file 1**. **Supplementary table 1.** Search string conducted on Pubmed/Medline. **Supplementary table 2.** Data extraction tool. **Supplementary table 3.** Adapted version of Newcastle-Ottawa score. **Supplementary table 4.** Data extraction summary of selected studies. **Supplementary table 5.** Meta-aggregation results of initial symptoms by subtypes of IIM. Factors identified in case studies as related to diagnostic delay. **Supplementary table 6.** Factors identified in case studies as related to diagnostic delay. **Supplementary table 7.** Factors of diagnostic delay by myositis types. **Supplementary figure 1.** Adapted version of Newcastle-Ottawa score. **Supplementary figure 2.** Contour-Enhanced funnel plot for mean diagnostic delay in diagnosis (n = 19). **Supplementary figure 3.** Forrest plot for mean diagnostic delay in all studies reporting standard deviation (no = SD not estimated, yes = SD estimated). **Supplementary figure 4.** Forrest plot for mean diagnostic delay in MSA tested and not tested studies. **Supplementary figure 5.** forrest plot for mean diagnostic delay in Peter Bohan's criteria and ENMC criteria. **Supplementary figure 6.** Forrest plot for mean diagnostic delay in multidisciplinary and specialist centres. Review protocol: Diagnostic delay of myositis: a protocol of an integrated systematic review.

## Data Availability

All data relevant to the study will be available upon reasonable request from the corresponding author.

## Abbreviations

IIM: Idiopathic inflammatory myositis
IBM: Inclusion body myositis
MSA: Myositis specific autoantibodies
DM: Dermatomyositis
PM: Polymyositis
IMNM: Immune-mediated necrotising myositis
NM: Necrotising myositis
JM: Juvenile myositis
JDM: Juvenile dermatomyositis
AMD: Amyopathic dermatomyositis
ASS: Anti-synthetase syndrome
ILD: Interstitial lung disease
EULAR: European League Against Rheumatism
ACR: The American College of Rheumatism
EULAR/ACR: European League Against Rheumatism/the American College of Rheumatism
PRISMA: Preferred reporting items for systematic reviews and meta-analyses
SD: Standard deviation
HR: Hazard ratio
