# Supplementary material for: Diagnostic delay of myositis: an integrated systematic review
Source: Orphanet J Rare Dis. 2022 Nov 21;17:420. doi: 10.1186/s13023-022-02570-9 (PMC9677896; doi:10.1186/s13023-022-02570-9)
Supplement: Supplementary file 1 — Additional file 1. Supplementary table 1. Search string conducted on Pubmed/Medline. Supplementary table 2. Data extraction tool. Supplementary table 3. Adapted version of Newcastle-Ottawa score. Supplementary table 4. Data extraction summary of selected studies. Supplementary table 5. Meta-aggregation results of initial symptoms by subtypes of IIM. Factors identified in case studies as related to diagnostic delay. Supplementary table 6. Factors identified in case studies as related to diagnostic delay. Supplementary table 7. Factors of diagnostic delay by myositis types. Supplementary figure 1. Adapted version of Newcastle-Ottawa score. Supplementary figure 2. Contour-Enhanced funnel plot for mean diagnostic delay in diagnosis (n = 19). Supplementary figure 3. Forrest plot for mean diagnostic delay in all studies reporting standard deviation (no = SD not estimated, yes = SD estimated). Supplementary figure 4. Forrest plot for mean diagnostic delay in MSA tested and not tested studies. Supplementary figure 5. forrest plot for mean diagnostic delay in Peter Bohan's criteria and ENMC criteria. Supplementary figure 6. Forrest plot for mean diagnostic delay in multidisciplinary and specialist centres. Review protocol: Diagnostic delay of myositis: a protocol of an integrated systematic review. [file 13023_2022_2570_MOESM1_ESM.docx]

**Diagnostic delay of Myositis: an integrated systematic review**

**Supplementary files**

**Supplementary table 1.**

| Supplementary table 1. Search string conducted on PUBMED/MEDLINE | | |
| --- | --- | --- |
| Search number | Query | Search Details |
| 1 | myositis[Title/Abstract] | "myositis"[Title/Abstract] |
| 2 | "delay in diagnosis"[Title/Abstract] | "delay in diagnosis"[Title/Abstract] |
| 3 | "diagnostic delay"[Title/Abstract] | "diagnostic delay"[Title/Abstract] |
| 4 | "misdiagnosis"[Title/Abstract] | "misdiagnosis"[Title/Abstract] |
| 5 | "time to diagnosis"[Title/Abstract] | "time to diagnosis"[Title/Abstract] |
| 6 | "incorrect diagnosis"[Title/Abstract] | "incorrect diagnosis"[Title/Abstract] |
| 7 | "missed diagnosis"[Title/Abstract] | "missed diagnosis"[Title/Abstract] |
| 8 | "delayed diagnosis"[Title/Abstract] | "delayed diagnosis"[Title/Abstract] |
| 9 | “slow diagnosis"[Title/Abstract] | “slow diagnosis"[Title/Abstract] |
| 10 | #2 OR #3 OR #4 OR #5 OR #6 OR #7 OR #8 OR #9 | "delay in diagnosis"[Title/Abstract] OR "diagnostic delay"[Title/Abstract] OR "misdiagnosis"[Title/Abstract] OR "time to diagnosis"[Title/Abstract] OR "incorrect diagnosis"[Title/Abstract] OR "missed diagnosis"[Title/Abstract] OR "delayed diagnosis"[Title/Abstract] |
| 11 | #1 AND #10 | "myositis"[Title/Abstract] AND ("delay in diagnosis"[Title/Abstract] OR "diagnostic delay"[Title/Abstract] OR "misdiagnosis"[Title/Abstract] OR "time to diagnosis"[Title/Abstract] OR "incorrect diagnosis"[Title/Abstract] OR "missed diagnosis"[Title/Abstract] OR "delayed diagnosis"[Title/Abstract]) |

**Supplementary table 2.**

| Supplementary table 2. Data extraction tool | |
| --- | --- |
| n | Data item |
| General information | |
| 1 | Study ID |
| 2 | Study title |
| 3 | Lead author name |
| 4 | Corresponding author’s contact details |
| 5 | Journal name |
| 6 | Publication date |
| 7 | Country in which the study was conducted |
| Methods | |
| 8 | Aim of the study |
| 9 | Study design |
| 10 | Study start date |
| 11 | Study end date |
| 12 | Study duration/Years covered (years) |
| 13 | Possible conflict of interest for study authors |
| 14 | Participant population description |
| 15 | Method of recruitment of participants |
| 16 | Inclusion criteria |
| Characteristics of study population | |
| 17 | Total number of participants |
| 18 | Total number of male participants |
| 19 | Total percent of male participants |
| 20 | Total number of female participants |
| 21 | Total percentage of female participants |
| 22 | Mean age of total participants at the recruitment |
| 23 | Mean age of male participants at recruitment |
| 24 | Mean age of female participants at recruitment |
| 25 | Mean age of total participants at symptom's onset (years) |
| 26 | Mean age of male participants at symptom’s onset (years) |
| 27 | Mean age of female participants at symptom's onset (years) |
| 28 | Mean age of total participants at correct diagnosis (years) |
| 29 | Mean age of male participants at correct diagnosis (years) |
| 30 | Mean age of female participants at correct diagnosis (years) |
| 31 | Diagnostic criteria used |
| 32 | IBM diagnosis (N) |
| 33 | IBM diagnosis (%) |
| 34 | DM diagnosis (N) |
| 35 | DM diagnosis (%) |
| 36 | PM diagnosis (N) |
| 37 | PM diagnosis (%) |
| 38 | IMNM diagnosis (%) |
| 39 | IMNM diagnosis (N) |
| 40 | Juvenile myositis (N) |
| 41 | Juvenile myositis (%) |
| 42 | ASS diagnosis (N) |
| 43 | ASS diagnosis (%) |
| 44 | Other myositis diagnosis (N) |
| 45 | Other myositis diagnosis (%) |
| Outcome 1. Diagnostic delay | |
| 46 | Total participants'/case groups' mean delay in diagnosis (estimate, months) |
| 47 | Total participants'/case groups' mean delay in diagnosis (SD) |
| 48 | Control groups' mean delay in diagnosis (estimate) |
| 49 | Control groups' mean delay in diagnosis (SD) |
| Outcome 2. Symptoms | |
| 50 | Description of initial symptom |
| 51 | Quote of initial symptom in the article |
| 52 | Initial symptoms start date |
| 53 | Action taken after the initial symptom |
| 54 | Diagnosis after the initial symptom/visit |
| 55 | Other relevant associated with initial symptom |
| 56 | Description of symptom that led to correct diagnosis |
| 57 | Quote of symptom that led to correct diagnosis in the article |
| 58 | Start date of symptom that led to correct diagnosis |
| 59 | Action taken after the symptom that led to correct diagnosis |
| 60 | Diagnosis after the symptom that led to correct diagnosis |
| 61 | Other relevant associated with the symptom that led to the correct diagnosis |
| 62 | Description of other relevant symptom or complains |
| 63 | Quote of other relevant symptom or complains in the article |
| 64 | Start date of other relevant symptom or complains |
| 65 | Action taken after the other relevant symptom or complains |
| 66 | Diagnosis after the other relevant symptom or complains |
| 67 | Other relevant associated with the other symptoms |
| Outcome 3. Treatment associated with delayed/incorrect diagnosis | |
| 68 | Main treatment after incorrect diagnosis |
| 69 | Number of total participants with main treatment after incorrect diagnosis |
| 70 | Percent of total participants with main treatment after incorrect diagnosis |
| Outcome 4. Factors associated with delay in diagnosis | |
| 71 | Description of a main factor associated with the delay in diagnosis/incorrect diagnosis |
| 72 | Quote of a main factor associated with the delay in diagnosis/incorrect diagnosis |
| 73 | Description of other factor associated with the delay in diagnosis/incorrect diagnosis |
| 74 | Quote of other factor associated with the delay in diagnosis/incorrect diagnosis |
| 75 | Other relevant factors of delay in diagnosis |
| Outcome 5. Experiences associated with delay in diagnosis | |
| 76 | Description of an experience in delayed diagnosis |
| 77 | Quote of an experience in delayed diagnosis |
| 78 | Description of other experience in delayed diagnosis |
| 79 | Quote of other experience in delayed diagnosis |
| 80 | Other relevant experiences |

**Supplementary table 3. Newcastle-Ottawa score**

| **Supplementary table 3. Adapted version of Newcastle-Ottawa score** | | | | | |
| --- | --- | --- | --- | --- | --- |
| Short reference | Representativeness of exposed cohort† | Disease definition‡ | Sample size justification* | Ascertainment of delay⁑ | Total score§ |
| Williams et al 2003., | 0 | 2 | 2 | 2 | 6 |
| Wargula et al 2001., | 0 | 2 | 2 | 1 | 5 |
| Triplett et al 2020., | 0 | 2 | 2 | 1 | 5 |
| Sayers et al 1992., | 0 | 2 | 2 | 1 | 5 |
| Rotar et al 2017., | 0 | 0 | 2 | 0 | 2 |
| Pijnenburg et al 2017., | 1 | 0 | 2 | 0 | 3 |
| Phillips et al 2000., | 1 | 2 | 2 | 0 | 5 |
| Paltiel et al 2015., | 0 | 0 | 2 | 0 | 2 |
| Needham et al 2008., | 0 | 2 | 2 | 1 | 5 |
| Munshi et al 2006., | 0 | 1 | 2 | 0 | 3 |
| Mathiesen et al 2010., | 2 | 2 | 2 | 1 | 7 |
| Lynn et al 2005., | 2 | 2 | 2 | 1 | 7 |
| Kucuksen et al 2012., | 0 | 1 | 2 | 1 | 4 |
| Kazamel et al 2016., | 0 | 2 | 2 | 1 | 5 |
| Hom et al 2019., | 0 | 1 | 2 | 0 | 3 |
| Herath et al 2018., | 0 | 2 | 2 | 0 | 4 |
| Felice et al 2001., | 1 | 2 | 2 | 1 | 6 |
| Dobloug et al 2015., | 1 | 2 | 2 | 1 | 6 |
| Dickison et al 2019., | 0 | 1 | 2 | 0 | 3 |
| Devi et al 2016., | 0 | 1 | 2 | 0 | 3 |
| De Langhe et al 2015., | 0 | 1 | 2 | 0 | 3 |
| Da Silva et al 2018., | 0 | 1 | 2 | 1 | 4 |
| Cobo-Ibanez et al 2019., | 0 | 2 | 2 | 1 | 5 |
| Chilingaryan et al 2015., | 0 | 1 | 2 | 0 | 3 |
| Cavagna et al 2015., | 1 | 2 | 2 | 1 | 6 |
| Badrising et al 2000., | 1 | 2 | 2 | 1 | 6 |
| Baccaro et al 2020., | 0 | 2 | 2 | 1 | 5 |
| †Representativeness (multi/national=1, single centre/survey=0))  ‡Disease definition (classification criteria=2, physician diagnosis=1, self-reported/unclear=0)  *Sample Size justification (yes=1, no=2)  ⁑Ascertainment of delay (interviews=2, review of records=1, not specified=0)  §Higher scores represent better quality | | | | | |

**Supplementary table 4. Data extraction summary of selected studies**

| **Supplementary table 4. Data extraction summary of selected studies categorized according to study designs** | | | | | | | | | | | | | | | | | |
| --- | --- | --- | --- | --- | --- | --- | --- | --- | --- | --- | --- | --- | --- | --- | --- | --- | --- |
| **Author** | **Country** | **MSA†** | **Study sample (n)** | **Mean age** | **IIM type** | **Initial specialist** | **Criteria used** | **Tissue biopsy** | **Mean delay**  **(months)** | **Mean delay SD^⁂^ (months)** | **Serum CK level** | **Initial/presenting symptoms (a) and main symptom (b)** | **Symptom that changed the diagnosis** | | **Factors related to delayed diagnosis** | **Experience/Outcomes of diagnostic delay** | **Treatment centre** |
| **Retrospective cohort studies** | | | | | | | | | | | | | | | | | |
| Baccaro et al 2020., | Brazil | yes | 55 | Not reported | ASS | Not reported | Criteria proposed by Connors et al and Cavagna et al (1, 2) | Not reported | 29 | 8.99 | Not reported | a. 41.8% of the cases had fever,  43.6% had joint symptoms, 38.2% had  myositis, 36.4% had interstitial lung disease,18.2% had Raynaud’s phenomenon,  and 16.4% had mechanic’s hands.  b. Not reported | Not reported | | 1. Subsequent clinical symptoms of ASS emerging at different timepoints | 1. Incorrect diagnosis  (Rheumatoid arthritis, non-specific interstitial  pneumopathy, or idiopathic pulmonary  interstitial fibrosis). | Multidisciplinary centre: Rheumatology department |
| Cobo-Ibanez et al 2019., | Spain | yes | 478 | 47.7 | All types of IIM | Not reported | Peter and Bohan’s criteria | Not reported | 3.48 | 7.85 | Not reported | a. Not reported  b. Muscle weakness (93.8% ), arthralgia/arthritis (63%), systemic manifestations (56%), Raynaud’s phenomenon (46.5%), dysphagia (35.7%), Mechanic’s hand (33.1%), DM rash (Gottron’s papules, Heliotrope rash, Gottron’s signs in 22% - 26%). | Not reported | | Not reported | 1. Delay in diagnosis was associated with mortality (HR 1.29, 95% CI 1.06-1.56) in IIM associated with interstitial lung disease | Multidisciplinary centre: Rheumatology department |
| Triplett et al 2020., | United States | yes | 67 | Not reported | NM | Not reported | Biopsy and electromyography | yes | 8.5 | 28.04 | Mean CK was not reported but CK>1000 was one of the best predictor of IMNM. | a. Not reported  b. Not reported | Not reported | | Not reported | Not reported | Multidisciplinary centre: Department of neurology |
| **Time series with comparison group** | | | | | | | | | | | | | | | | | |
| Sayers et al 1992., | United States | no | 32 | 61 | IBM | Not re[ported | Peter and Bohan’s criteria | yes | 34 | 24.7 | 1524 U/L (22/32 cases) | a. Not reported  b. Symmetric proximal muscle weakness in 29 of 32 cases | Not reported | | Not reported | Not reported | Multidisciplinary centre: Department of immunologic and rheumatologic diseases |
| **Analytical cross-sectional studies or time series** | | | | | | | | | | | | | | | | | |
| Cavagna et al 2015., | Multi-national | yes | 44 | 53.5 | ASS | Not reported | Clinical characteristics and positive anti Jo-1 | Not reported | 5 | 6.51 | Not reported | a. Arthritis (37%), myositis (40%), interstitial lung disease (58%), isolated arthritis (15.5%), isolated ILD (32.5%), isolated myositis (23%), fever (27%), mechanic’s hands (22%), Raynaud’s phenomenon (43%)  b. Arthritis (48%), myositis (52%), interstitial lung disease (83%), fever (30.5%), mechanic’s hands (27%), Raynaud’s phenomenon (45%) | Not reported | | Not reported | Not reported | Not reported |
| Kazamel et al 2016., | United States | no | 51 | Not reported | IBM | Not reported | Grigg's pathological criteria (3) | yes | 74.4 | 75.67 | Not reported | a. Muscle weakness  b. Upper limb muscle weakness | Not reported | | Not reported | Not reported | Multidisciplinary centre: Department of neurology |
| Mathiesen et al 2010., | Denmark | no | 57 | Not reported | JDM | Not reported | Peter and Bohan’s criteria | yes | 8 | 1.6 | 1744 U/L (54 cases) | a. Proximal muscle weakness (93%), fatigue (82%), myalgia (75%), myalgia (75%), nonspecific skin rash (75%), Gottron’s papules (74%), Heliotrope rash (67%), arthralgia (40%), periungual capillary changes (35%), weight loss (33%), fever (30%), vascular changes including Raynaud’s phenomenon (28%), arthritis (26%), contractures (26%), gastrointestinal complaints (25%), dysphagia (23%), infection (18%), muscle atrophy (16%), skin ulceration (16%), dyspnoea (11%), dysphonia (11%), calcinosis (5%), lipodystrophy (4%)  b. Proximal muscle weakness | Not reported | | 3. Physician’s unawareness of the condition | 4. Worse outcome as short disease duration was correlated with less organ damage | Not reported |
| Pijnenburg et al 2017., | France | no | 40 | 48.2 | All types of IIM | Not reported | ENMC criteria | yes | 16.4 | 4.5 | Dropped head syndrome- 2795 U/L  Other IIM- 2865 U/L | a. Not reported  b. Proximal muscle weakness | Not reported | | Not reported | Worse outcome as camptocormia and dropped head syndrome are associated  with late onset scleromyositis and sIBM with delayed diagnosis | Specialist centres: French Myositis Network and the Club Rhumatisme et Inflammation |
| Wargula et al 2001., | United States | no | 59 | 7.9 | JDM | Not reported | Peter and Bohan’s criteria | yes | 5.3 | 5.2 | Not reported | a. Skin symptoms including Gottron’s papules, heliotrope’s rash, periorbital oedema (22-88%), nailfold capillary changes (756%), malar erythema (48%), periungual erythema (46%), arthralgia (61%), weight loss (48%), fatigue (44%), abdominal pain (37%), dysphagia (31%),muscle pain (31%), fever (29%), dyspnoea (31%), palpitations (5%), melena (3%)  b. Proximal muscle weakness (100%), Gottron’s papules (93%), heliotrope rash (71%) | Not reported | | Not reported | Not reported | Multidisciplinary centre |
| Williams et al 2003., | Australia | no | 13 | 68 | All types of IIM | Not reported | Serum creatinine kinase level, electromyography, and biopsy | yes (8/13 cases had bipsy results) | 55 | 53.49 | >200 U/L (6 out of 8 cases tested) | a. Dysphagia (9/13) and limb or facial weakness (1/3)  b. Not reported | Not reported | 7. Absence of muscle weakness in presentation of dysphagia  8. False negative rate of laboratory test including creatinine kinase, ESR and ANA | | Not reported | Multidisciplinary centre |
| **Non-comparative studies (descriptive cross-sectional studies, survey and prevalence or incidence studies)** | | | | | | | | | | | | | | | | | |
| Badrising et al 2000., | The Netherlands | no | 76 | Not reported | IBM | Not reported | ENMC criteria of 1997 | yes | 96 | 71.24 | Not reported | a. Not reported  b. Not reported | Not reported | | Not reported | 6. Misdiagnosis as motor neuron disease, myopathy or polyneuropathy | Other: Rheumatology and neurology centres |
| Da Silva et al 2018., | United States | no | 232 | Not reported | DM | Not reported | Not reported | Not reported | 15.5 | 46.61 | Not reported | a. Not reported  b. Skin lesion (86%-92.6%): Gottron's sign, V sign, shawl sign, holster sign, heliotrope, mechanics hands, and periungual, telangiectasia, erythema, or dystrophy | Not reported | | Not reported | 2. Misdiagnosis as lupus or undifferentiated connective tissue disease | Multidisciplinary centre: Department of dermatology |
| Dobloug et al 2015., | Norway | yes | 100 | Not reported | IBM | Not reported | ENMC criteria of 1997 or 2011 | yes | 67.2 | 60 | Mean CK level – 804 U/L | a. Not reported  b. Muscle weakness (proximal and knee extension weakness in all patients and most had distal muscle weakness e.g. finger flexor weakness) | Not reported | | Not reported | Not reported | Multidisciplinary centre |
| Felice et al 2001., | United States | no | 35 | 70 | IBM | Neurologist (74%), rheumatologist, general internist and orthopaedic surgeon | Definite or possible IBM as proposed by Griggs et al (3) | yes | 68.4 | 68.47 | Mean CK- 444 U/L; | a. Muscle weakness (Leg weakness– difficulty rising from a chair or ascending/descending stairs, facial weakness, hand grip weakness) and dysphagia  b. Not reported | Not reported | | 2. Resemblance to motor neuron disease | 3. Misdiagnosis as motor neuron disease, unspecified myopathy, facioscapulohumeral muscular dystrophy, oculopharyngeal muscular dystrophy and peripheral nerve diseases | Specialist centres: Muscular Dystrophy Association Clinic |
| Lynn et al 2005., | New Zealand | no | 6 | Not reported | IBM | Not reported | Peter and Bohan’s criteria/Mastaglia and Phillips (4) | yes | 43.2 | 10.3 | Mean CK- 3589 U/L | a. Proximal muscle weakness (the most common), distal weakness, rash, dysphagia, dyspnoea, myalgia, arthralgia  b. Not reported | Not reported | | Not reported | Not reported | Multidisciplinary centre |
| Needham et al 2008., | Australia | no | 57 | Not reported | IBM | Not reported | Needham and Mastaglia’s criteria (5) | yes | 62.4 | 39.24 | Not reported | a. Not reported  b. Not reported | Not reported | | 4. Mistaking symptoms to normal ageing due to lack of awareness by an individual  5. Failure to recognise the cardinal histological changes in the biopsy  6. Selection of inappropriate muscle to biopsy | 5. Misdiagnosis as arthritis, motor neurone disease and ‘‘old age” | Specialist centres: Australian Neuromuscular Research Institute and other rheumatology and neurology specialists |
| Paltiel et al 2015., | Multi-national | no | 280 | 70.4 | IBM | Not reported | Not reported | yes | 56.4 | Not reported | Not reported | a. Muscle weakness (falling, tripping, difficulty walking, climbing stairs), fatigue and trouble swallowing  b. Not reported | Not reported | | Not reported | 7. Misdiagnosis as arthritis and Polymyositis in cases of IBM  8. Worse outcome for every year since diagnosis, there was an average decrease of 0.38 in the overall functional index (P<0.0001), highlighting disease  progression and impairment | Not reported |
| Phillips et al 2000., | Australia | no | 17 | Not reported | IBM | Not reported | Grigg's pathological criteria (3) | yes | 52.8 | 26.72 | Not reported | a. Not reported  b. Not reported | Not reported | | Not reported | Not reported | Specialist centres: Australian Neuromuscular Research Institute |
| Rotar et al 2017., | Slovenia | no | 79 | Not reported | All types of IIM | Not reported | Records of biopsy | yes | 6.66 | 6.77 | Not reported | a. Myositis, skin rash, weight loss, lung symptoms, arthritis, fever  b. Not reported | Not reported | | Not reported | Not reported | Multidisciplinary centre: Department of rheumatology |
| **Non-comparative studies (Case reports)** | | | | | | | | | | | | | | | | | |
| De Langhe et al 2015., | Belgium | yes | 1 | 44 | ASS | Respiratory specialist | Clinical characteristics and presence of antisynthetase antibodies | no | 48 | Not calculatable | 713 U/L | a. dyspnoea,  Raynaud’s phenomenon and subtle swelling of fingers and eyelids.  b. Not reported | 1. Symptoms progressed: dyspnoea, fever, night sweats, weight loss, myalgia, muscle weakness, arthralgia, swollen eyelids and cracked fingers | | 9. Lung symptoms presenting before other features | 9. Misdiagnosis as undifferentiated connective tissue disease | Multidisciplinary centre: Department of pulmonology (respiratory) |
| Devi et al 2016., | India | yes | 1 | 35 | ASS | Respiratory specialist | Not reported | no | 0 | Not calculatable | Normal | a. Cough, breathlessness, fever  b. Not reported | 2. No response to treatment | | Not reported | 10. Misdiagnosed as pneumonia  12. Incorrect treatment with antibiotics | Other: Department of pulmonology (respiratory) |
| Dickison et al 2019., | United States | yes | 1 | 31 | DM | Not reported | Clinical characteristics and biopsy | yes | 120 | Not calculable | Not reported | a. Vulvovaginitis  b. Not reported | 3. New symptom: Dermatological signs such as shawl sign, rash, erythematous patches | | 10. Rare presentation of dermatomyositis | 12. Misdiagnosed as bacterial vaginitis  13. Incorrect medical treatment | Other: Gynaecologist and dermatologist |
| Herath et al 2018., | Sri Lanka | yes | 1 | 53 | DM | Not reported | Peter and Bohan’s criteria | yes | 5 | Not calculable | 100 U/L | a. Fever and rash  b. Not reported | 4. Symptoms progressed: extensive poikiloderma of up to 90% of the skin  5. New symptom: appearance if heliotrope rash, shawl sign and Gottron papules | | 11. Rare manifestation of dermatomyositis | 14. Incorrect medical treatment with fluids, steroids, and antibiotics  15. Early discharge | Multidisciplinary centre |
| Hom et al 2019., | United States | no | 1 | 58 | IBM | Not reported | Biopsy | yes | 60 | Not calculable | 90-142U/L | a. Dysphagia, muscle weakness, weight loss that continued for 8 years  b. Not reported | 6. Symptoms progressed: loss of weight to BMI 14 | | 12. Broad differential diagnosis for weakness | 16. IIM was not diagnosed but Sjorgen’s syndrome was diagnosed correctly  17. Progression of symptoms: loss of weight to BMI 14 | Multidisciplinary centre |
| Kucuksen et al 2012., | Turkey | no | 1 | 63 | IBM | Not reported | Biopsy | yes | 60 | Not calculatable | 529 U/L | a. Slowly progressive muscle weakness (painless) and atrophy that continued for 5 years  b. Not reported | 7. Symptoms progressed: need of assistance in daily activities | | Not reported | 18. Progression of symptoms: patient needed assistance in daily activities | Multidisciplinary centre |
| Munshi et al 2006., | UK | no | 1 | 81 | IBM | Not reported | Biopsy | yes | 36 | Not calculatable | 677-996U/L | a. Muscle weakness  b. Not reported | 8. Symptoms progressed: recurrent falls and need of assistance in daily activities | | 13. Muscle weakness was not regarded as GP attributed it to residual deficits from stroke. | 19. Progression of symptoms: recurrent falls | Multidisciplinary centre |
| **Non-comparative studies (Case series)** | | | | | | | | | | | | | | | | | |
| Chilingaryan et al 2015., | United States | no | 20 | 67.8 | IBM | Case 1- Neurologist  Case 2- Not reported | Not reported | 18/20 cases had muscle biopsy | 70 | 54.8 | Case 1 – 647 IU/L  Case 2 – 2400 IU/L | a. In 20 cases all reported muscle weakness from grip weakness to difficulty getting up from chair  b. Not reported | 9. Symptoms progressed: inability to get up, walk and climb stairs | | 14. Overlapping symptoms  15. Mixed electrodiagnostic findings  16. Atypical symptoms  17. Overreliance on electrophysiological  18 not enough findings on muscle biopsy | 20. Misdiagnosed as ALS, entrapment neuropathy, Parkinson's  21. Incorrect treatments for ALS, tunnel release and Parkinson's | Multidisciplinary centre (Neuromuscular division) |
| **†**MSA- Myositis specific antibody test  **^⁂^**SD- Standard deviation | | | | | | | | | | | | | | | | | |

**Supplementary figure 1. Adapted version of Newcastle-Ottawa scale
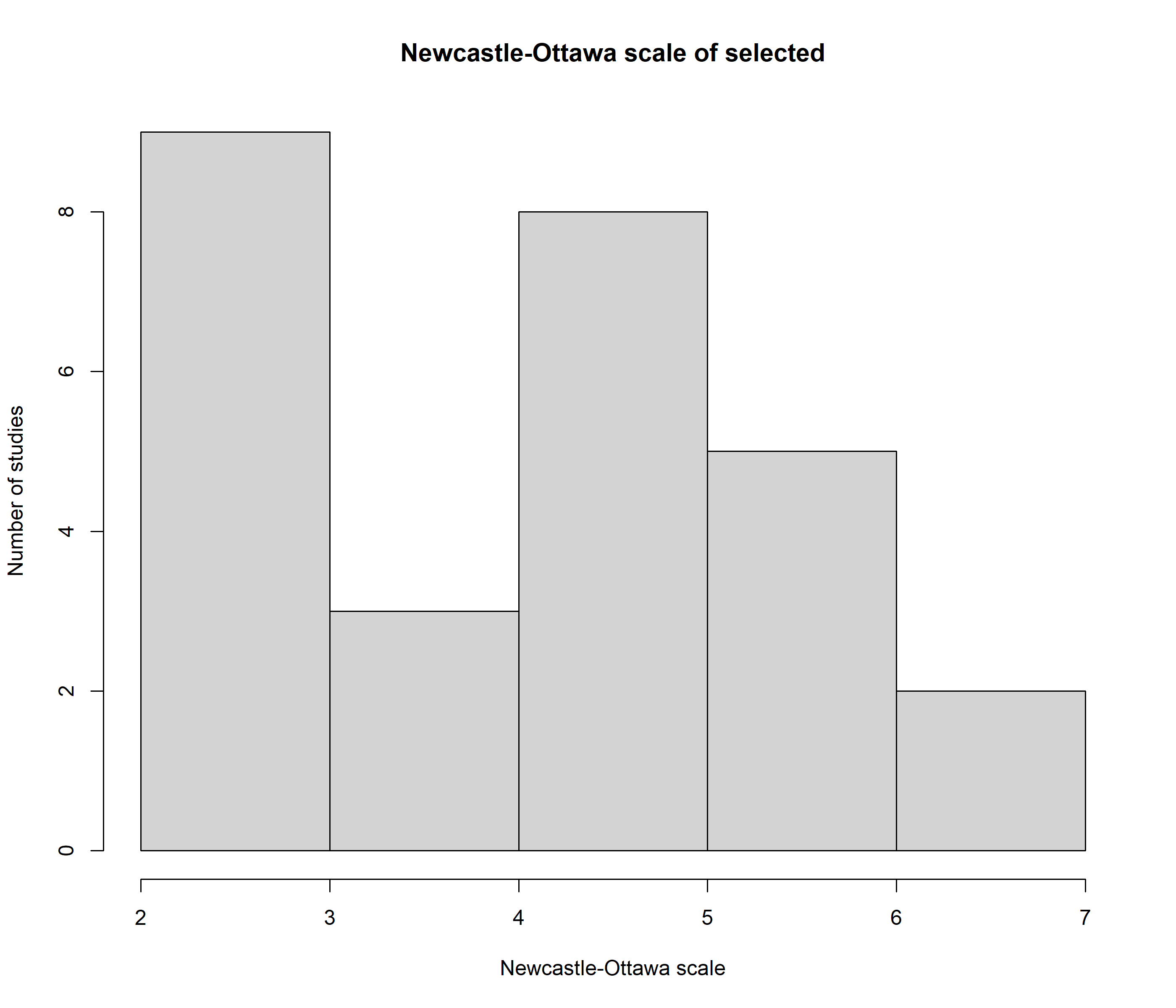
**

**Supplementary table 5.**

| **Supplementary table 5. Meta-aggregation results of initial symptoms by subtypes of IIM** | | |
| --- | --- | --- |
| **Author** | **Initial symptoms** | **Meta-aggregated initial symptoms** |
| **ASS** | | |
| **Retrospective cohort studies** | | |
| Baccaro et al 2020., | Fever (41.8%) | 1. General symptoms |
|  | Joint symptoms (43.6%), | 2. Joint associated symptoms |
|  | Myositis (38.2%), | 3. Muscle associated symptoms |
|  | Interstitial lung disease (36.4%), | 4. Lung symptoms |
|  | Raynaud’s phenomenon (18.2%), | 5. Connective tissue disease associated symptoms |
|  | Mechanic’s hands (16.4%). | 5. Skin symptoms |
| **Analytical cross-sectional studies or time series** | | |
| Cavagna et al 2015., | Arthritis (37%), | 2. Joint associated symptoms |
|  | Myositis (40%), | 3. Muscle associated symptoms |
|  | Interstitial lung disease (58%), | 4. Lung symptoms |
|  | Isolated arthritis (15.5%), | 2. Joint associated symptoms |
|  | Isolated ILD (32.5%), | 4. Lung symptoms |
|  | Isolated myositis (23%), | 3. Muscle associated symptoms |
|  | Fever (27%), | 1. General symptoms |
|  | Mechanic’s hands (22%), | 5. Skin symptoms |
|  | Raynaud’s phenomenon (43%) | 5. Connective tissue disease associated symptoms |
| **Non-comparative studies (Case reports)** | | |
| De Langhe et al 2015., | Dyspnoea, | 4. Lung symptoms |
|  | Raynaud’s phenomenon, | 5. Connective tissue disease associated symptoms |
|  | Subtle swelling of fingers and eyelids. | 5. Skin symptoms |
| Devi et al 2016., | Cough, | 4. Lung symptoms |
|  | Breathlessness/dyspnea, | 4. Lung symptoms |
|  | Fever | 1. General symptoms |
| **DM** | | |
| **Non-comparative studies (Case reports)** | | |
| Dickison et al 2019., | Vulvovaginitis | 5. Skin symptoms |
| Herath et al 2018., | Fever, | 1. General symptoms |
|  | Rash | 5. Skin symptoms |
| **IBM** | | |
| **Time series with comparison group** | | |
| Kazamel et al 2016., | Muscle weakness | 3. Muscle associated symptoms |
| **Non-comparative studies (descriptive cross-sectional studies, survey and prevalence or incidence studies)** | | |
| Felice et al 2001., | Muscle weakness (Leg weakness– difficulty rising from a chair or ascending/descending stairs, facial weakness, hand grip weakness) | 3. Muscle associated symptoms |
|  | Dysphagia | 3. Muscle associated symptoms |
| Lynn et al 2005., | Proximal muscle weakness (the most common), | 3. Muscle associated symptoms |
|  | Distal weakness, | 3. Muscle associated symptoms |
|  | Rash, | 5. Skin symptoms |
|  | Dysphagia, | 3. Muscle associated symptoms |
|  | Dyspnoea, | 3. Muscle associated symptoms |
|  | Myalgia, | 3. Muscle associated symptoms |
|  | Arthralgia | 2. Joint associated symptoms |
| Paltiel et al 2015., | Muscle weakness (falling, tripping, difficulty walking, climbing stairs), | 3. Muscle associated symptoms |
|  | Fatigue | 1. General symptoms |
|  | Trouble swallowing | 3. Muscle associated symptoms |
| **Non-comparative studies (Case reports)** | | |
| Hom et al 2019., | Dysphagia, | 3. Muscle associated symptoms |
|  | Muscle weakness, | 3. Muscle associated symptoms |
|  | Weight loss | 1. General symptoms |
| Kucuksen et al 2012., | Slowly progressive muscle weakness (painless) | 3. Muscle associated symptoms |
|  | Muscle atrophy | 3. Muscle associated symptoms |
| Munshi et al 2006., | Muscle weakness | 3. Muscle associated symptoms |
| **Non-comparative studies (Case series)** | | |
| Chilingaryan et al 2015., | In 20 cases all reported muscle weakness from grip weakness to difficulty getting up from chair | 3. Muscle associated symptoms |
| **JDM** | | |
| **Time series with comparison group** | | |
| Mathiesen et al 2010., | Proximal muscle weakness (93%), | 3. Muscle associated symptoms |
|  | Fatigue (82%), | 1. General symptoms |
|  | Myalgia (75%), | 3. Muscle associated symptoms |
|  | Nonspecific skin rash (75%), | 5. Skin symptoms |
|  | Gottron’s papules (74%), | 5. Skin symptoms |
|  | Heliotrope rash (67%), | 5. Skin symptoms |
|  | Arthralgia (40%), | 2. Joint associated symptoms |
|  | Periungual capillary changes (35%), | 5. Skin symptoms |
|  | Weight loss (33%), | 1. General symptoms |
|  | Fever (30%), | 1. General symptoms |
|  | Vascular changes including Raynaud’s phenomenon (28%), | 5. Connective tissue disease associated symptoms |
|  | Arthritis (26%), | 2. Joint associated symptoms |
|  | Contractures (26%), | 3. Muscle associated symptoms |
|  | Gastrointestinal complaints (25%), | 6. Other |
|  | Dysphagia (23%), | 3. Muscle associated symptoms |
|  | Infection (18%), | 1. General symptoms |
|  | Muscle atrophy (16%), | 3. Muscle associated symptoms |
|  | Skin ulceration (16%), | 5. Skin symptoms |
|  | Dyspnoea (11%), | 4. Lung symptoms |
|  | Dysphonia (11%), | 3. Muscle associated symptoms |
|  | Calcinosis (5%), | 6. Other |
|  | Lipodystrophy (4%) | 6. Other |
| **Analytical cross-sectional studies or time series** | | |
| Wargula et al 2001., | Skin symptoms including Gottron’s papules, Heliotrope’s rash, periorbital oedema (22-88%), | 5. Skin symptoms |
|  | Nailfold capillary changes (75.6%), | 5. Skin symptoms |
|  | Malar erythema (48%), | 5. Skin symptoms |
|  | Periungual erythema (46%), | 5. Skin symptoms |
|  | Arthralgia (61%), | 2. Joint associated symptoms |
|  | Weight loss (48%), | 1. General symptoms |
|  | Fatigue (44%), | 1. General symptoms |
|  | Abdominal pain (37%), | 6. Other |
|  | Dysphagia (31%), | 3. Muscle associated symptoms |
|  | Muscle pain/myalgia (31%), | 3. Muscle associated symptoms |
|  | Fever (29%), | 1. General symptoms |
|  | Dyspnoea (31%), | 4. Lung symptoms |
|  | Palpitations (5%), | 4. Lung symptoms |
|  | Melena (3%) | 6. Other |
| **NM** | | |
| **Not reported** | | |
| **All types of IIM** | | |
| **Analytical cross-sectional studies or time series** | | |
| Williams et al 2003., | Dysphagia (69.2%), | 3. Muscle associated symptoms |
|  | Limb or facial weakness (33%) | 3. Muscle associated symptoms |
| **Non-comparative studies (descriptive cross-sectional studies, survey and prevalence or incidence studies)** | | |
| Rotar et al 2017., | Myositis, | 3. Muscle associated symptoms |
|  | Skin rash, | 5. Skin symptoms |
|  | Weight loss, | 1. General symptoms |
|  | Lung symptoms, | 4. Lung symptoms |
|  | Arthritis, | 2. Joint associated symptoms |
|  | Fever | 1. General symptoms |
|  |  |  |

**Supplementary table 6.**

| Supplementary table 6. Factors identified in case studies as related to diagnostic delay. | |
| --- | --- |
| Level of factor | Factor |
| Health care service | Mixed electrodiagnostic findings that can confuse clinicians (6)  False negative of laboratory tests including normal serum creatinine kinase levels, ESR and ANA(7) |
| Clinician | Lack of clinician’s awareness of IBM symptoms (8, 9)  Failure to recognise histological changes of IBM (9)  Failure to select appropriate muscle for biopsy (9)  Failure to recognize muscle weakness due to co-morbidities, such as residuals of stroke (10) |
| Complex clinical characteristics of IIM | Symptoms emerging at different timepoints(6)  Atypical symptoms, such as camptocormia or foot drop, or an incomplete clinical picture(6)  Broad differential diagnosis for muscle weakness(11)  Rare presentation of IIM and lung presentation before other features (12)  Resemblance of motor neuron disease(13)  Overlapping symptoms with non-IBM diseases(14)  Rare presentation of DM (15, 16)  Absence of muscle weakness in presentation of dysphagia(7) |
| Individual level factor | Individuals mistaking muscle weakness as normal ageing(9) |

**Supplementary table 7.**

| **Supplementary table 7.** Factors of diagnostic delay by myositis types | | | |
| --- | --- | --- | --- |
| **Author** | **Factors** | **Meta – aggregation of factors** | |
| **ASS** | | | |
| **Retrospective cohort studies** | | | |
| Baccaro et al 2020., | 1. Subsequent clinical symptoms of ASS emerging at different timepoints | 1. Complex clinical characteristics of ASS  (1 factor) | |
| **Analytical cross-sectional studies or time series** | | |  |
| **Non-comparative studies (Case reports)** | | | |
| De Langhe et al 2015., | 2. Lung symptoms presenting before other features | 1. Complex clinical characteristics of ASS  (1 factor) | |
| **DM** | | |  |
| **Non-comparative studies (Case reports)** | | | |
| Dickison et al 2019., | 17. Rare presentation of dermatomyositis | 1. Complex clinical characteristics of IIM  (2 factors) | |
| Herath et al 2018., | 18. Rare manifestation of dermatomyositis |  |  |
| **IBM** | | | |
| **Non-comparative studies (descriptive cross-sectional studies, survey and prevalence or incidence studies)** | | | |
| Felice et al 2001., | 5. Resemblance to motor neuron disease | 1. Complex clinical characteristics of IIM  (1 factor) | |
| Needham et al 2008., | 6. Mistaking symptoms to normal ageing due to lack of awareness by an individual | 2. Clinician related factors  (3 factors) | |
|  | 7. Failure to recognise the cardinal histological changes in the biopsy |  |  |
|  | 8. Selection of inappropriate muscle to biopsy |  |  |
| **Non-comparative studies (Case reports)** | | | |
| Hom et al 2019., | 9. Broad differential diagnosis for weakness | 1. Complex clinical characteristics of IIM  (1 factor) | |
| Munshi et al 2006., | 10. Muscle weakness was not regarded as GP attributed it to residual deficits from stroke. | 2. Clinician related factors  (1 factor) | |
| **Non-comparative studies (Case series)** | |  | |
| Chilingaryan et al 2015., | 11. Overlapping symptoms | 1. Complex clinical characteristics of IBM  (3 factors) | |
|  | 12. Mixed electrodiagnostic findings |  |  |
|  | 13. Atypical symptoms |  |  |
|  | 14. Overreliance on electrophysiological | 2. Clinician related factors  (1 factor) | |
|  | 15. Not enough findings on muscle biopsy | 1. Complex clinical characteristics of IBM  (1 factor) | |
| **JDM** | | | |
| **Time series with comparison group** | | | |
| Mathiesen et al 2010., | 16. Physician’s unawareness of the condition | 2. Clinician related factors  (1 factor) | |
| **All types of IIM** | | |  |
| **Retrospective cohort studies** | | | |
| Williams et al 2003., | 3. Absence of muscle weakness in presentation of dysphagia | 1. Complex clinical characteristics of IIM  (1 factor) | |
|  | 4. False negative rate of laboratory test including creatinine kinase, ESR and ANA | 2. Health care service | |

**Supplementary figure 2. Contour-Enhanced funnel plot for mean diagnostic delay in diagnosis (n=19)
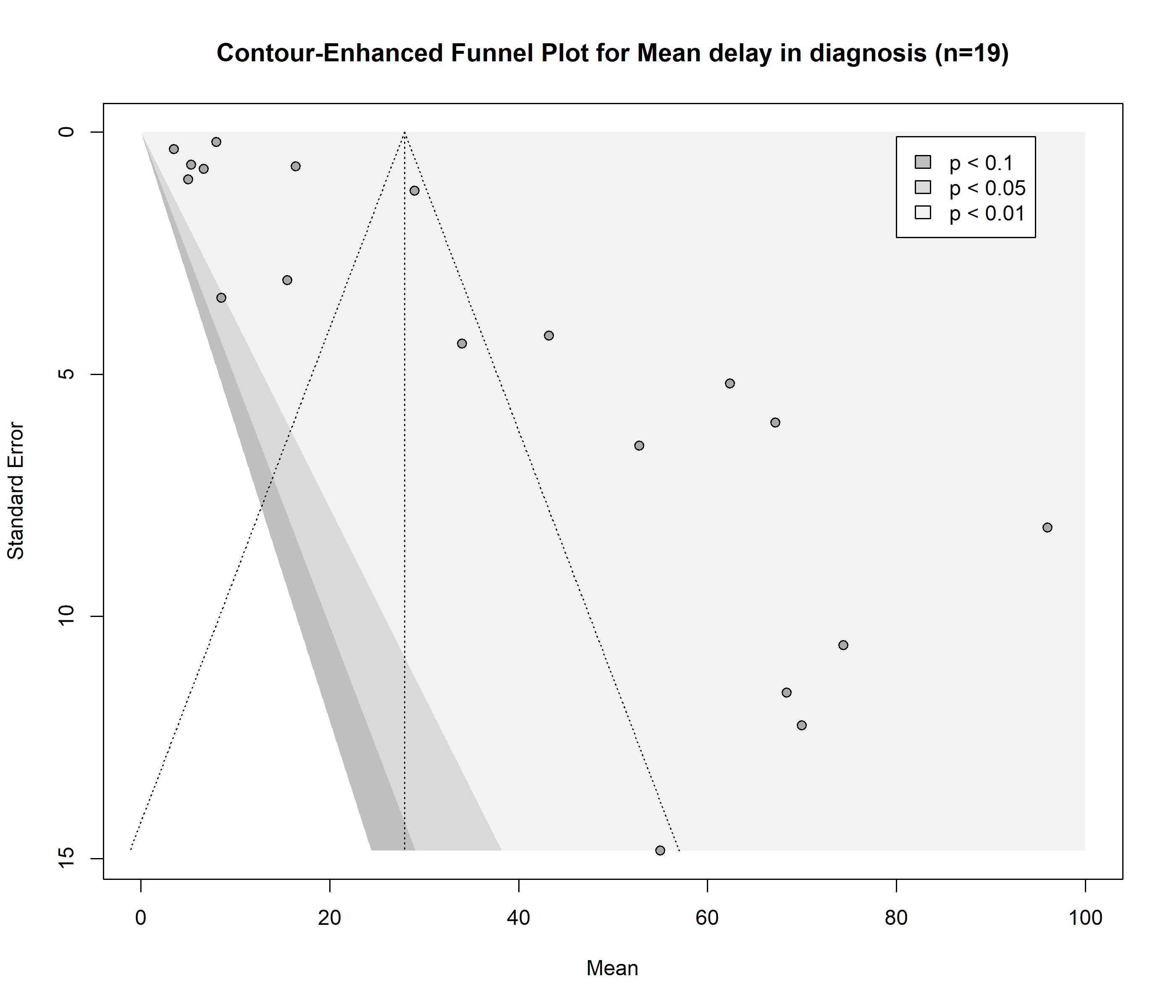
**

**Supplementary figure 3. Forrest plot for mean diagnostic delay in all studies and studies reporting standard deviation (no=SD not estimated, yes=SD estimated)**

**
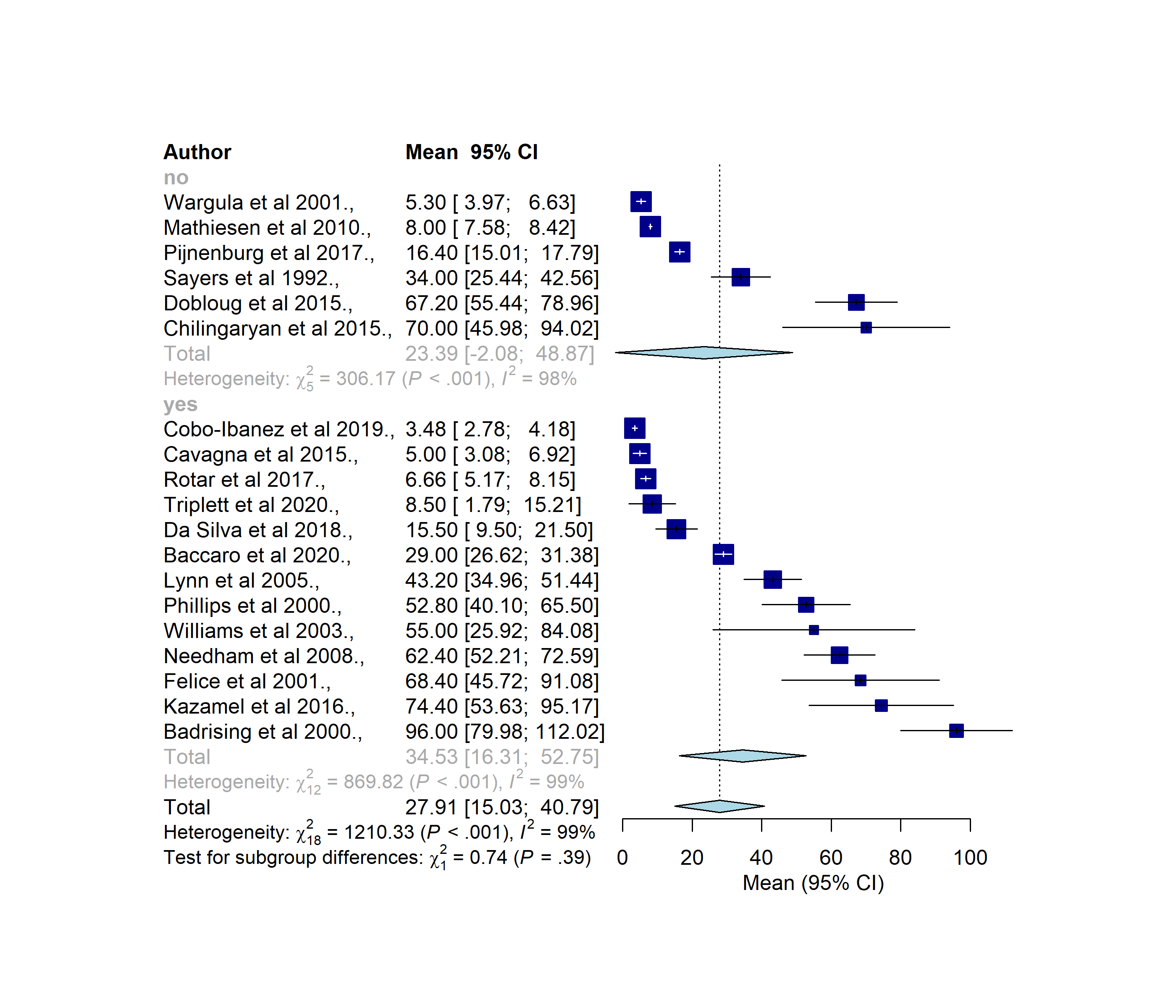
**

**Supplementary figure 4. Forrest plot for mean diagnostic delay in MSA tested and not tested studies**

**
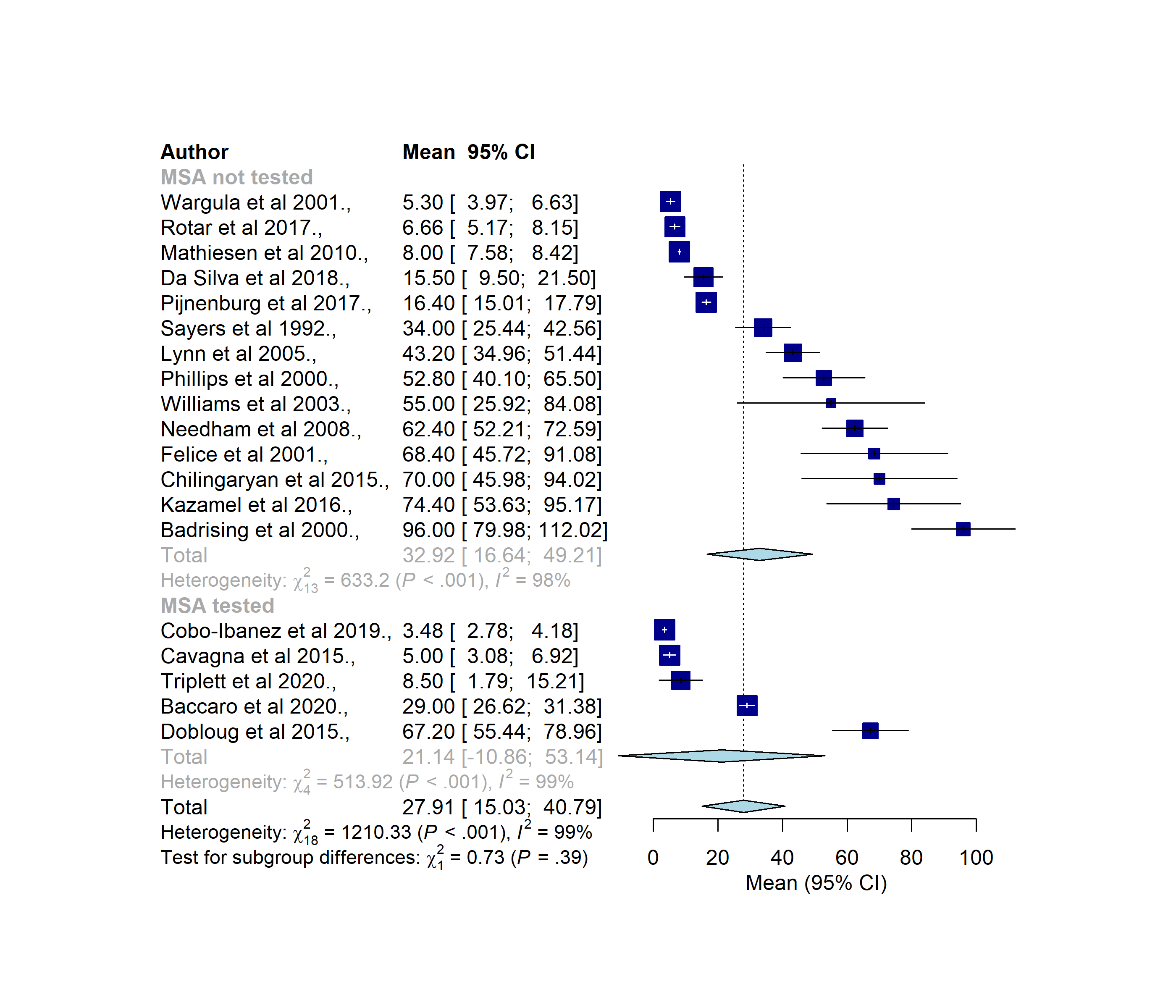
**

**Supplementary figure 5. Forrest plot for mean diagnostic delay in Peter Bohan’s criteria and ENMC criteria**

**
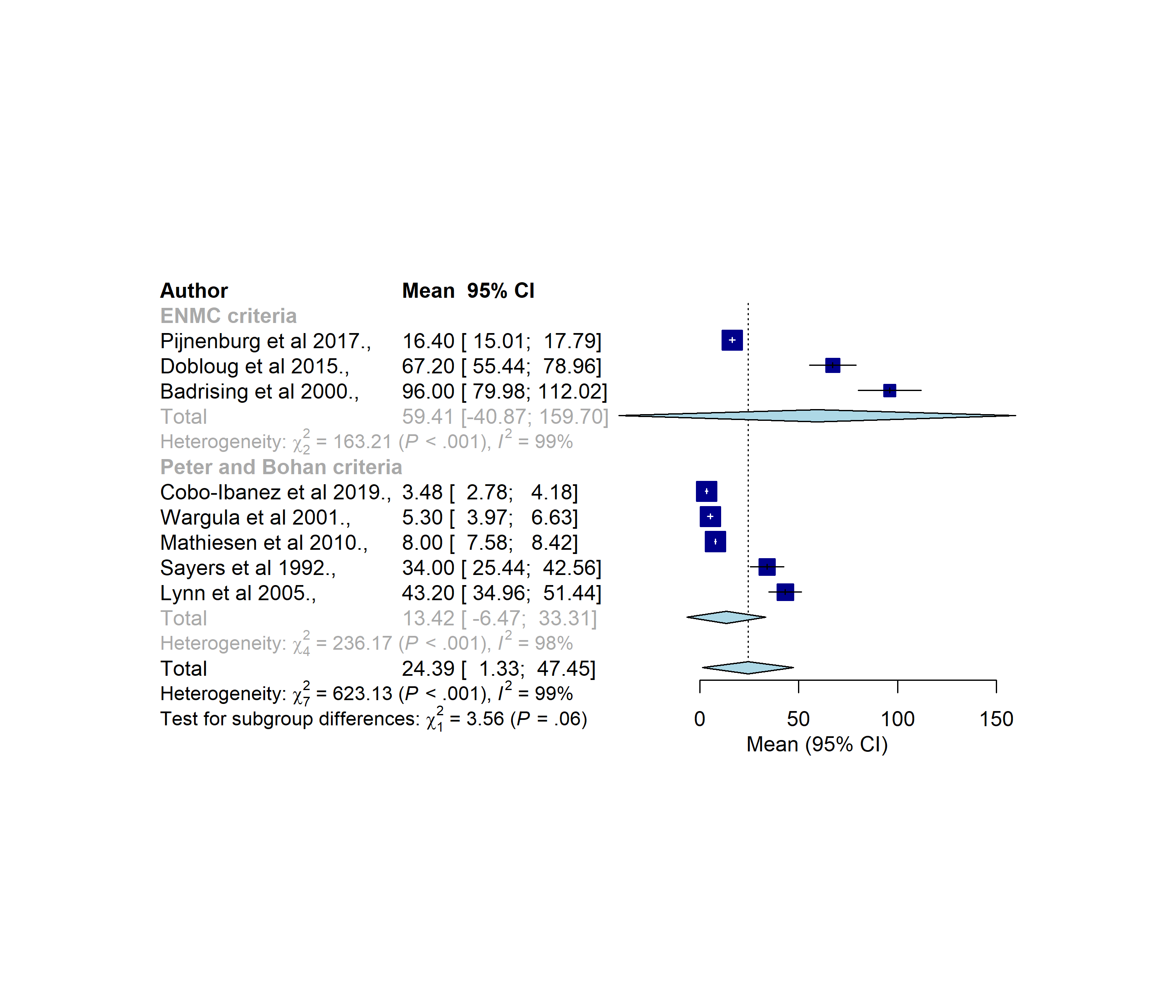
Supplementary figure 6. Forrest plot for mean diagnostic delay in multidisciplinary and specialist centres**

**
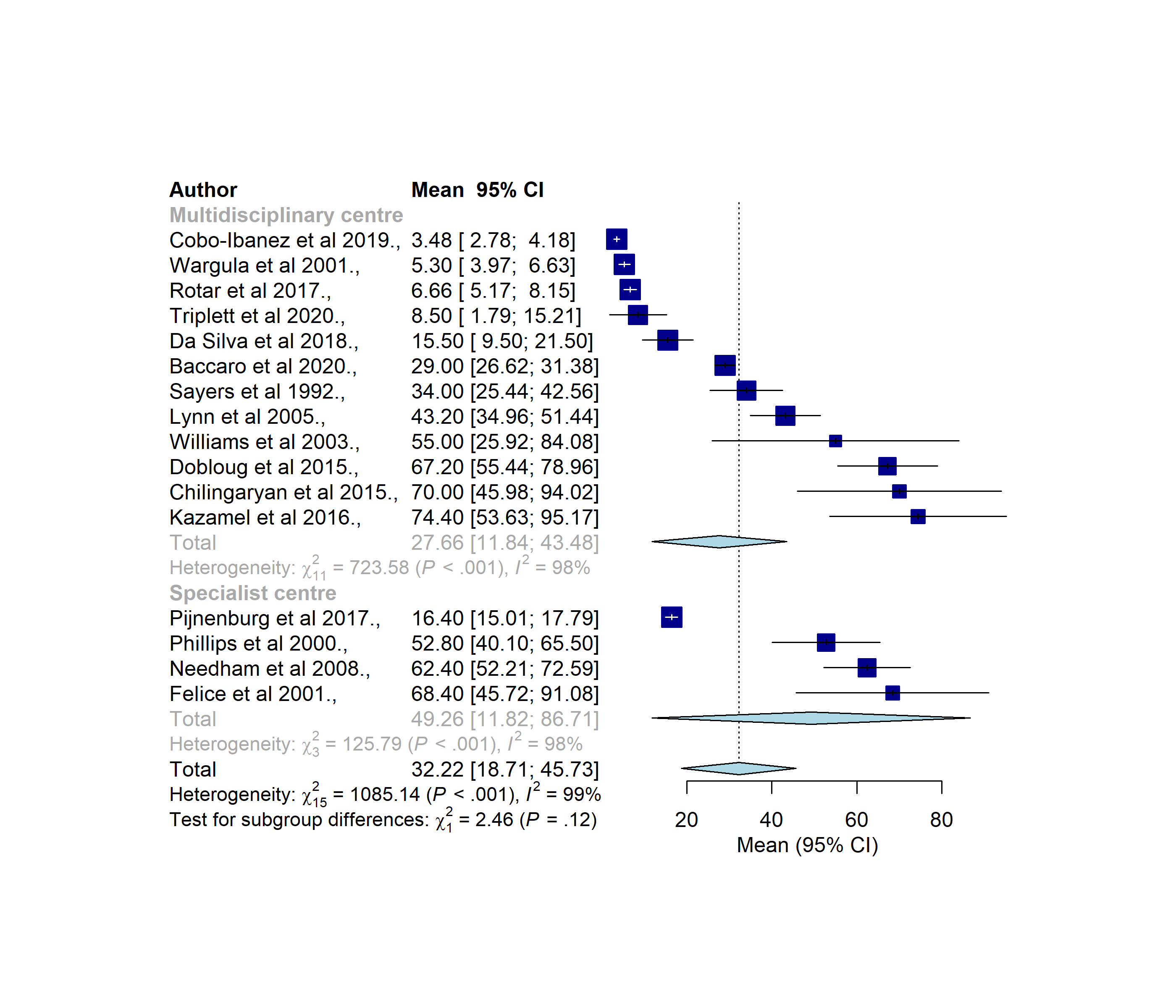
**

**Review’s protocol**

Diagnostic delay of Myositis: a protocol of an integrated systematic review

# Section 1: Administration Information

## Item 1. Title

Diagnostic delay of Myositis: a protocol of an integrated systematic review

## Item 2. Registration

This systematic review will be registered with PROSPERO.

## Item 3. Authors

Tergel Namsrai MD, MSc^1^

Jane Desborough RN, RM, MPH, PhD^1^*

Anita Chalmers OAM^1,2^

Christine Lowe^1,2^

Matthew Cook MBBS, PhD, FRACP, FRCPA, FFSc(RCPA)^3^

Christine Phillips MBBS, BMedSc, MA, MPH, DipEd, FRACGP, MD, AM^4^

Anne Parkinson BA (Hons), AFHEA, PhD^1^

### Item 3a. Affiliations

1. National Centre for Epidemiology and Population Health, Australian National University,

Canberra, Australia

2. The Myositis Association- Australia Inc

3. John Curtin School of Medical Research, Australian National University, Canberra, Australia

4. Australian National University Medical School, Canberra, Australia

### *Correspondence

Jane Desborough, National Centre for Epidemiology and Population Health, Australian National University, 63, Eggleston Road, Acton ACT, 2601, Australia

Email: [Jane.Desborough@anu.edu.au](mailto:Jane.Desborough@anu.edu.au)

### Item 3b. Contributions

TN drafted the review protocol. All authors will contribute to the study. TN and AP are the primary reviewers. JD is the third reviewer guarantor of the study.

## Item 4. Amendments

In the event of protocol amendments, date, explanation, and rationale of the amendment will be described in this section. The record will be in tabular format as shown below.

| Table1. Record of Amendments | | | | |
| --- | --- | --- | --- | --- |
| Date | Section | Original protocol | Revised protocol | Rationale |
| 09/12/  2021 | Appendix 1. Search string | "myositis"[Title/Abstract] AND ("delay in diagnosis"[Title/Abstract] OR "diagnostic delay"[Title/Abstract] OR "misdiagnosis"[Title/Abstract] OR "time to diagnosis"[Title/Abstract] OR "incorrect diagnosis"[Title/Abstract] OR "missed diagnosis"[Title/Abstract] OR "delayed diagnosis"[Title/Abstract]) | "myositis"[Title/Abstract] AND ("delay in diagnosis"[Title/Abstract] OR "diagnostic delay"[Title/Abstract] OR "misdiagnosis"[Title/Abstract] OR "time to diagnosis"[Title/Abstract] OR "incorrect diagnosis"[Title/Abstract] OR "missed diagnosis"[Title/Abstract] OR "delayed diagnosis"[Title/Abstract]) OR “slow diagnosis”[Title/Abstract]) | After peer review “slow diagnosis” was added to the search terms. The search string has changed accordingly. |

## Item 5. Support

### Item 5a. Sources

This integrated systematic review is part of the “Missed opportunities in clinical practice: Tools to enhance healthcare providers’ awareness and diagnosis of rare diseases in Australia” project funded by the Commonwealth represented by the Department of Health Australia (Grant ID 4-G5ZN0T7).

### Item 5b and 5c. Sponsor name and its role

The Commonwealth of Australia represented by the Department of Health has provided a grant for the “Missed opportunities in clinical practice: Tools to enhance healthcare providers’ awareness and diagnosis of rare diseases in Australia” project which includes this review.

# Section 2: Introduction

## Item 6. Rationale

Diagnostic delay of Myositis: a protocol of an integrated systematic review

Idiopathic inflammatory myopathies (IIM) commonly described as “inflammatory myositis”, are a heterogenous group of rare muscular autoimmune diseases of muscle inflammation initially presenting with asymmetric distal muscle weaknesses (finger flexor and knee flexor muscles) progressing over time to larger muscle weaknesses (gluteus, quadriceps and throat muscles) and other extra muscular features such as skin manifestations (17).

There are several subtypes of IIM including dermatomyositis (DM), polymyositis (PM), Inclusion body myositis (IBM) and other specified idiopathic myositis (i.e. immune-mediated necrotizing myopathy (IMNM), juvenile myositis (JM), juvenile dermatomyositis (JDM), amyopathic dermatomyositis (AMD) and anti-synthetase syndrome (ASS)), and unspecified idiopathic inflammatory myositis (18).

IIM is characterized as a rare disease as its prevalence is relatively low compared to other disorders. A recent systematic review of 16 articles reported an overall estimated incidence rate of 78 cases/100,000 per year for IIM (19).

However, IIM has broad clinical characteristic features involving both muscular and extra-muscular systems with acute or progressive onset. In addition to general muscle features it can present with dysphagia (39%), lung involvement causing interstitial lung disease (ILD) (30%), malignancy (13%), and cardiac disease (9%) (20).

There has been significant and promising progress on Myositis Specific Autoantibodies in the last decade. The presence of these antibodies assists the suspected diagnosis of IIM (21). Additionally, MRI imaging can reveal specific changes in the involved muscle and therefore aids the diagnostic process of IIM (22). However, there is a lack of any conclusive diagnostic test and commonly used comprehensive diagnostic criteria. The most widely used criteria is Bohan and Peter’s criteria which recognizes PM and DM as IIM (23). Later, Dalakas introduced different criteria which take into account AMD (24). However, these two criteria both still exclude IBM as an individual type of IIM.

In 2018, the European League Against Rheumatism (EULAR) and the American College of Rheumatism (ACR), developed diagnostic and classification criteria based on the data from 976 IIM cases and 624 comparators (25). The EUCLAR/ARC criteria permit specialists to differentiate between all possible IIM subgroups that are not mentioned in previously used criteria, including JM, JDM and IMNM.

Due to the low prevalence, broad range of clinical features, lack of conclusive diagnostic testing and comprehensive globally accepted criteria, timely diagnosis of IIM can be challenging and result in significant diagnostic delays. Some studies reported diagnostic delay of 4-5.6 years in cases of IBM. However, studies examining the overall diagnostic delay, contributing factors, and people’s experience in IIM are scarce. Further studies are crucial for gaining clearer insight into diagnostic delays. This will inform future studies, interventions, tools, and health policies directed at enhancing diagnostic efficiency and patient experience of Myositis.

## Item 7. Objectives

The aim of this integrated systematic review is to review the evidence regarding diagnostic delay in Myositis. To this end, the review will answer the following questions:

- 1. What are the causes and consequences of diagnostic delay of Myositis?
  2. What evidence is there about patients’ experience of diagnostic delay of Myositis?

# Section 3: Methods

## Item 8. Eligibility criteria

The studies will be selected according to the eligibility criteria developed using the PICOS tool (26)

Inclusion criteria:

- 1. Participants

We will include all studies examining people of all ages with myositis including dermatomyositis, polymyositis, necrotizing myositis, juvenile dermatomyositis, inclusion body myositis, mixed connective tissue diseases, overlap myositis, interstitial myositis, orbital myositis and antisynthetase syndrome.

- 1. Exposure

We will include all studies examining delayed, incorrect, or missed diagnosis of Myositis (outlined above in section 1 Participants).

- 1. Comparison or control group

Given the aim of the study we will not include a control group.

- 1. Outcome of interest

The main outcomes of interest are time to diagnosis, factors associated with diagnostic delay and patients’ experiences of diagnosis of Myositis.

We will include quantitative studies with adequately reported data (the actual words of the participant or the field notes of observers) as well as findings (the results of the researcher’s analysis and interpretation).

We will include qualitative studies with patients’ experience of diagnostic delay for Myositis.

- 1. Timing

There will be no restriction in timing of the studies.

- 1. Setting

There will be no restriction in settings.

- 1. Study design

We will include all types of study design such as observational studies, clinical trials, case-reports, and qualitative studies, except for review articles. However, the reference lists of review articles will be hand searched for relevant papers.

- 1. Language

We will include studies published in English, Indonesian and German.

## Item 9. Information sources

Electronic database and grey literature searches will be conducted.

1. Electronic database searches: PUBMED/MEDLINE, Scopus, and ProQuest.
2. Other methods to identify relevant literature: grey literature will be searched using Google Scholar.

The search strategy will be developed using the PICOS method as recommended in the Cochrane systematic review handbook (26).

## Item 10. Search strategy

The search strategy was developed to ensure reproducibility and increase transparency following the PRISMA-P checklist (27). Research questions and search terms were developed using the PICOS tool (Population/Intervention/Comparison/Outcomes/Study Design) to enhance the scientific literature by ensuring reliability and homogeneity of search results (26).

The primary source of literature will be a systematic search of multiple electronic databases (from inception onwards): PubMed/Medline, Scopus, and ProQuest. Sources of grey literature will also be searched. A search of the grey literature will be conducted through Open Access Theses and Dissertation (<https://oatd.org/> ), ProQuest thesis and dissertations, The National Library of Australia, and The Myositis Association Australia website (<https://myositis.org.au/> ). Additionally, reference lists of selected studies and review articles will be searched. All settings and study design will be considered.

Search terms were developed in collaboration with research team members and peer reviewed (TN, AP, JD, MC, CP) using the PRESS checklist (28). Search terms were combined using Boolean operators “AND” and “OR”. Preliminary exploratory searches of the literature were undertaken (15 October 2021) to inform the final search strategy and determine outcomes. The final search strategy that was developed and used on PUBMED/MEDLINE database is shown in Appendix 1.

## Item 11. Study records

### Item 11a. Data management

The literature search results will be imported to Covidence, an internet-based software that facilitates collaboration between reviewers and ensures independent review of the literature (29)

### Item 11b. Selection process

Two review authors will independently screen the titles and abstracts of literature identified in the search against the pre-developed inclusion criteria (TN and AP). Any conflict in the title and abstract screening process will be discussed among the review team and will be resolved by a third reviewer (JD). Full reports for all studies that meet the inclusion criteria or where there is any uncertainty will be obtained. Review authors will then screen full text reports according to the inclusion criteria. The reasons for excluding studies will be recorded. Authors will not be blinded to the study types, journals, and authors during this process.

### Item 11c. Data collection

After the study selection process is complete, a data extraction tool will be designed, peer reviewed and piloted. In the piloting process, two independent reviewers will extract data independently and in duplicate from five studies each and compare their results to establish agreement and validity of the data extraction tool. Any disagreements will be resolved through discussion and conflicts resolved by a third reviewer (JD). We will contact study authors to resolve any uncertainties about extracted data.

## Item 12. Data items

The following data items will be extracted:

Identification of the study

Journal,

Authors,

Year,

Citation,

Research center/university/hospital/organization,

Conflict of interest,

Funding/sponsorship.

Methods

Study aim,

Study design,

Participant demographics,

Recruitment process,

Inclusion,

Exclusion criteria,

Statistical analysis.

Main findings

Exposure details,

Diagnostic delays,

Factors associated with diagnostic delay,

Patients’ experience,

Relevant outcomes.

## Item 13. Outcomes and prioritization

Primary outcome

Diagnostic delay time (time from symptom onset to correct diagnosis) in people living with Myositis

Secondary outcomes

Patient’s experiences related to diagnostic delay

Causes and consequences of diagnostic delay

## Item 14. Quality assessment or risk of bias

The selected studies will be assessed for methodological quality or risk of bias using the Mixed Methods Appraisal Tool (MMAT) designed to critically appraise mixed method studies included in systematic reviews (30). Two independent review authors will conduct the quality appraisal. Any conflicts will be resolved with discussion and a third reviewer’s vote (JD).

## Item 15. Data synthesis

A systematic narrative synthesis will be undertaken to explore the findings of included studies in relation to time from symptom onset to diagnosis, and people’s experiences related to delayed diagnosis in line with guidance from the Centre for Reviews and Dissemination (31).

If extracted quantitative data are homogenous, a meta-analysis will be conducted using a random-effects model. Extracted qualitative data will be meta-synthesized using meta-aggregation. In line with meta-aggregation methods, findings (processed data) from qualitative studies will be extracted and aggregated into a single set of categories, which will then be further aggregated and synthesised into a set of statements that are meaningful for clinical practice.

Further methods and stages of meta-analysis will be discussed if collected data is quantitively synthesizable. The findings from the quantitative and qualitative studies will be reported separately; however, the discussion will be integrative of both.

## Item 16. Confidence in cumulative estimate

If a meta-analysis is conducted, the quality/certainty of evidence for all quantitative outcomes will be judged using the Grading of Recommendations Assessment, Development and Evaluation (GRADE) working group methodology (32). Certainty of the body of evidence will be assessed across the domains of risk of bias, consistency of effect, imprecision, indirectness, and publication bias. The certainty will be reported in four levels: high, moderate, low, and very low.

## Item 17. Timeline and stages of review

| Table 2. Timeline and process of systematic review | | | |
| --- | --- | --- | --- |
|  | Started | Completed | Timeline |
| Protocol development | Yes | Yes | November, 2021 |
| Search strategy development | Yes | Yes | November 2021 |
| Preliminary literature search | Yes | Yes | November, 2021 |
| Literature search | Yes | No | November, 2021 |
| Piloting of the study selection process | No | No | January, 2021 |
| Study selection | No | No | January-February 2022 |
| Quality appraisal | No | No | February, 2022 |
| Data extraction | No | No | January- February 2022 |
| Data synthesis | No | No | February- March, 2022 |
| Writing paper | No | No | March-May, 2022 |

## Version history

| Table 3. Version history of systematic review protocol | | |
| --- | --- | --- |
| Date | Version number | Explanation |
| 28 October 2021 | Version 1.0 | First draft of review, “Diagnostic delay of Myositis: a protocol of an integrated systematic review” |
| 25 November 2021 | Version V2.0 | Second draft of review, “Diagnostic delay of Myositis: a protocol of an integrated systematic review” |

# Appendix 1.

Search terms used to develop final search string for PubMed search conducted on 9th^th^ of December 2021.

## Search string:

"myositis"[Title/Abstract] AND ("delay in diagnosis"[Title/Abstract] OR "diagnostic delay"[Title/Abstract] OR "misdiagnosis"[Title/Abstract] OR "time to diagnosis"[Title/Abstract] OR "incorrect diagnosis"[Title/Abstract] OR "missed diagnosis"[Title/Abstract] OR "delayed diagnosis"[Title/Abstract])

## Search history

| Table 4. Search history of Myositis search for PUBMED/MEDLINE | | | | |
| --- | --- | --- | --- | --- |
| Search number | Query | | Search Details | Results |
| 11 | #1 AND #10 | | "myositis"[Title/Abstract] AND ("delay in diagnosis"[Title/Abstract] OR "diagnostic delay"[Title/Abstract] OR "misdiagnosis"[Title/Abstract] OR "time to diagnosis"[Title/Abstract] OR "incorrect diagnosis"[Title/Abstract] OR "missed diagnosis"[Title/Abstract] OR "delayed diagnosis"[Title/Abstract] OR "slow diagnosis"[Title/Abstract]) | 96 |
| 10 | #2 OR #3 OR #4 OR #5 OR #6 OR #7 OR #8 OR #9 | | "delay in diagnosis"[Title/Abstract] OR "diagnostic delay"[Title/Abstract] OR "misdiagnosis"[Title/Abstract] OR "time to diagnosis"[Title/Abstract] OR "incorrect diagnosis"[Title/Abstract] OR "missed diagnosis"[Title/Abstract] OR "delayed diagnosis"[Title/Abstract] | 38,185 |
| 9 | "slow diagnosis"[Title/Abstract] | | "slow diagnosis"[Title/Abstract] | 8 |
| 8 | "delayed diagnosis"[Title/Abstract] | | "delayed diagnosis"[Title/Abstract] | 8,589 |
| 7 | "missed diagnosis"[Title/Abstract] | "missed diagnosis"[Title/Abstract] | | 2,399 |
| 6 | "incorrect diagnosis"[Title/Abstract] | "incorrect diagnosis"[Title/Abstract] | | 1,299 |
| 5 | "time to diagnosis"[Title/Abstract] | "time to diagnosis"[Title/Abstract] | | 2,661 |
| 4 | "misdiagnosis"[Title/Abstract] | "misdiagnosis"[Title/Abstract] | | 16,610 |
| 3 | "diagnostic delay"[Title/Abstract] | "diagnostic delay"[Title/Abstract] | | 3,203 |
| 2 | "delay in diagnosis"[Title/Abstract] | "delay in diagnosis"[Title/Abstract] | | 5,967 |
| 1 | myositis[Title/Abstract] | "myositis"[Title/Abstract] | | 11,085 |

1. Imbert-Masseau A, Hamidou M, Agard C, Grolleau JY, Chérin P. Antisynthetase syndrome. Joint Bone Spine. 2003;70(3):161-8.

2. Cavagna L, Trallero-Araguás E, Meloni F, Cavazzana I, Rojas-Serrano J, Feist E, et al. Influence of Antisynthetase Antibodies Specificities on Antisynthetase Syndrome Clinical Spectrum Time Course. J Clin Med. 2019;8(11).

3. Griggs RC, Askanas V, DiMauro S, Engel A, Karpati G, Mendell JR, et al. Inclusion body myositis and myopathies. Ann Neurol. 1995;38(5):705-13.

4. Mastaglia FL, Phillips BA. Idiopathic inflammatory myopathies: epidemiology, classification, and diagnostic criteria. Rheum Dis Clin North Am. 2002;28(4):723-41.

5. Needham M, Mastaglia FL. Inclusion body myositis: current pathogenetic concepts and diagnostic and therapeutic approaches. Lancet Neurol. 2007;6(7):620-31.

6. Baccaro ACCD, Pinto GLB, Carboni RCS, Shinjo SK. The clinical manifestations at the onset of antisynthetase syndrome: A chameleon with multiple faces. Reumatismo. 2020;72(2):86-92.

7. Williams RB, Grehan MJ, Hersch M, Andre J, Cook IJ. Biomechanics, diagnosis, and treatment outcome in inflammatory myopathy presenting as oropharyngeal dysphagia. Gut. 2003;52(4):471-8.

8. Mathiesen PR, Zak M, Herlin T, Nielsen SM. Clinical features and outcome in a Danish cohort of juvenile dermatomyositis patients. Clinical and Experimental Rheumatology. 2010;28(5):782-9.

9. Needham M, Corbett A, Day T, Christiansen F, Fabian V, Mastaglia FL. Prevalence of sporadic inclusion body myositis and factors contributing to delayed diagnosis. J Clin Neurosci. 2008;15(12):1350-3.

10. Munshi SK, Thanvi B, Jonnalagadda SJ, Da Forno P, Patel A, Sharma S. Inclusion body myositis: an underdiagnosed myopathy of older people. Age Ageing. 2006;35(1):91-4.

11. Hom J, Marwaha S, Postolova A, Kittle J, Vasquez R, Davidson J, et al. A Patient with Sjogren’s Syndrome and Subsequent Diagnosis of Inclusion Body Myositis and Light-Chain Amyloidosis. Journal of General Internal Medicine. 2019;34(6):1058-62.

12. De Langhe E, Lenaerts J, Bossuyt X, Westhovens R, Wuyts WA. Mechanic's hands in a woman with undifferentiated connective tissue disease and interstitial lung disease - Anti-PL7 positive antisynthetase syndrome: A case report. Journal of Medical Case Reports. 2015;9(1).

13. Felice KJ, North WA. Inclusion body myositis in Connecticut: Observations in 35 patients during an 8-year period. Medicine. 2001;80(5):320-7.

14. Chilingaryan A, Rison RA, Beydoun SR. Misdiagnosis of inclusion body myositis: two case reports and a retrospective chart review. J Med Case Rep. 2015;9:169.

15. Herath H, Keragala B, Pahalagamage SP, Janappriya G, Kulatunga A, Gunasekera CN. Erythroderma and extensive poikiloderma - a rare initial presentation of dermatomyositis: a case report. J Med Case Rep. 2018;12(1):83.

16. Dickison S, Grither W, Compton L, Chibnall R, Jones H. Dermatomyositis Presenting as Vulvovaginitis. Obstetrics and Gynecology. 2019;134(2):409-12.

17. Malik A, Hayat G, Kalia JS, Guzman MA. Idiopathic Inflammatory Myopathies: Clinical Approach and Management. Frontiers in neurology. 2016;7:64-.

18. World Health Organization. International statistical classification of diseases and related health problems. 2019.

19. Meyer A, Meyer N, Schaeffer M, Gottenberg J-E, Geny B, Sibilia J. Incidence and prevalence of inflammatory myopathies: a systematic review. Rheumatology. 2014;54(1):50-63.

20. Lilleker J, Vencovsky J, Wang G, Wedderburn L, Diederichsen L, Schmidt J, et al. The EuroMyositis registry: an international collaborative tool to facilitate myositis research. Annals of the Rheumatic Diseases. 2017;77.

21. Satoh M, Tanaka S, Ceribelli A, Calise SJ, Chan EKL. A Comprehensive Overview on Myositis-Specific Antibodies: New and Old Biomarkers in Idiopathic Inflammatory Myopathy. Clinical reviews in allergy & immunology. 2017;52(1):1-19.

22. Maurer B, Walker UA. Role of MRI in diagnosis and management of idiopathic inflammatory myopathies. Curr Rheumatol Rep. 2015;17(11):67.

23. Bohan A, Peter JB. Polymyositis and dermatomyositis (first of two parts). N Engl J Med. 1975;292(7):344-7.

24. Dalakas MC, Hohlfeld R. Polymyositis and dermatomyositis. The Lancet. 2003;362(9388):971-82.

25. Bottai M, Tjärnlund A, Santoni G, Werth VP, Pilkington C, de Visser M, et al. EULAR/ACR classification criteria for adult and juvenile idiopathic inflammatory myopathies and their major subgroups: a methodology report. RMD Open. 2017;3(2):e000507.

26. Methley AM, Campbell S, Chew-Graham C, McNally R, Cheraghi-Sohi S. PICO, PICOS and SPIDER: a comparison study of specificity and sensitivity in three search tools for qualitative systematic reviews. BMC Health Services Research. 2014;14(1):579.

27. Moher D, Shamseer L, Clarke M, Ghersi D, Liberati A, Petticrew M, et al. Preferred reporting items for systematic review and meta-analysis protocols (PRISMA-P) 2015 statement. Systematic Reviews. 2015;4(1):1.

28. McGowan J, Sampson M, Salzwedel DM, Cogo E, Foerster V, Lefebvre C. PRESS Peer Review of Electronic Search Strategies: 2015 Guideline Statement. Journal of Clinical Epidemiology. 2016;75:40-6.

29. Veritas Health Innovation. Covidence systematic review software Melbourne, Australia [Available from: Available at [www.covidence.org](https://anu365-my.sharepoint.com/personal/u6468437_anu_edu_au/Documents/Health%20Experience%20RSHP/Rare%20disease%20project%202021-2023/Literature%20reviews/Myositis/Manuscript%20of%20Myositis%20review%20paper/Orphanet%20submission/www.covidence.org).

30. Hong QN, Fàbregues S, Bartlett G, Boardman F, Cargo M, Dagenais P, et al. The Mixed Methods Appraisal Tool (MMAT) version 2018 for information professionals and researchers. Education for Information. 2018;34:285-91.

31. Centre for Reviews and Dissemination. Systematic Reviews. University of York: York Publishing Services Ltd; 2009.

32. Balshem H, Helfand M, Schünemann HJ, Oxman AD, Kunz R, Brozek J, et al. GRADE guidelines: 3. Rating the quality of evidence. J Clin Epidemiol. 2011;64(4):401-6.
